# CRISPR-Cas9 Targeting of the *eIF4E1* Gene Extends the Potato Virus Y Resistance Spectrum of the *Solanum tuberosum* L. cv. Desirée

**DOI:** 10.3389/fmicb.2022.873930

**Published:** 2022-06-01

**Authors:** Alessandra Lucioli, Raffaela Tavazza, Simona Baima, Karoly Fatyol, Jozsef Burgyan, Mario Tavazza

**Affiliations:** ^1^Biotechnology Laboratory, Biotechnology and Agroindustry Division, Department for Sustainability, ENEA, CR Casaccia, Rome, Italy; ^2^Research Centre for Genomics and Bioinformatics, Council for Agricultural Research and Economics (CREA), Rome, Italy; ^3^Agricultural Biotechnology Institute, National Agricultural Research and Innovation Centre, Godollo, Hungary

**Keywords:** potato, translation initiation factors, eIF4E, genome editing, potyvirus resistance, PVY

## Abstract

Translation initiation factors and, in particular, the eIF4E family are the primary source of recessive resistance to potyviruses in many plant species. However, no eIF4E-mediated resistance to this virus genus has been identified in potato (*Solanum tuberosum* L.) germplasm. As in tomato, the potato eIF4E gene family consists of *eIF4E1*, its paralog *eIF4E2, eIF(iso)4E*, and *nCBP*. In tomato, *eIF4E1* knockout (KO) confers resistance to a subset of potyviruses, while the *eIF4E1/2* double KO, although conferring a broader spectrum of resistance, leads to plant developmental defects. Here, the tetraploid potato cv. Desirée owning the dominant *Ny* gene conferring resistance to potato virus Y (PVY) strain O but not NTN was used to evaluate the possibility to expand its PVY resistance spectrum by CRISPR-Cas9-mediated KO of the *eIF4E1* susceptibility gene. After a double process of plant protoplast transfection-regeneration, *eIF4E1* KO potatoes were obtained. The knockout was specific for the *eIF4E1*, and no mutations were identified in its *eIF4E2* paralog. Expression analysis of the eIF4E family shows that the disruption of the *eIF4E1* does not alter the RNA steady-state level of the other family members. The *eIF4E1* KO lines challenged with a PVY^NTN^ isolate showed a reduced viral accumulation and amelioration of virus-induced symptoms suggesting that the *eIF4E1* gene was required but not essential for its multiplication. Our data show that *eIF4E1* editing can be usefully exploited to broaden the PVY resistance spectrum of elite potato cultivars, such as Desirée, by pyramiding eIF4E-mediated recessive resistance.

## Introduction

Potato is a member of the *Solanaceae* family and, on a global scale, is the fourth most cultivated species as a food source right after the three main cereal crops: rice, wheat, and corn.^1^ Like other vegetatively propagated plants, potato is prone to diseases, and potato virus Y (PVY), the type member of the *Potyvirus* genus, is the most harmful pathogen affecting potato yield and quality (Valkonen, 1997, 2007; Kreuze et al., 2020).

The PVY genome consists of a 9.7 kb monopartite positive-sense single-stranded RNA (ssRNA) encoding 11 functional proteins, one of which the VPg is covalently linked to the 5′ end of the genome and is a virulence factor. PVY isolates can be grouped into at least seven strains (PVY^O^, PVY^C^, PVY^Z^, PVY^E^, PVY^N^, PVY^N–Wi^, and PVY^NTN^) based on their biological properties (Lacomme and Jacquot, 2017).

PVY is non-persistently transmitted by more than sixty aphid species (Lacomme and Jacquot, 2017). Since insecticides are ineffective to manage non-persistently transmitted viruses (Kirchner et al., 2014) the spread of PVY in the field is difficult to control unless resistant plants are used.

Two types of dominant PVY resistance have been described in potatoes, the hypersensitive response (HR) and the extreme resistance (ER) conferred by *N* (*Ny*, *Nc*, and *Nz*) and *Ry* (*Ryadg*, *Rysto*, and *Rycbc*) genes, respectively (Karasev and Gray, 2013). *N* genes were the first used in potato-breeding programs for PVY resistance; hence, most elite potato cultivars possess one or more *N* genes (Valkonen et al., 2017). *Ny*, *Nc*, and *Nz* genes confer strain-group-specific HR to virus isolates belonging to the PVY^O^, PVY^C^, and PVY^Z^ strains, respectively (Singh et al., 2008). Notably, recombinant PVY strains, such as the PVY^NTN^, inducing the potato tuber necrosis ringspot disease (PTRND) and overcoming all three *N* genes, have emerged and become prevalent in fields exposing well-accepted consumer potato cultivars to PVY disease (Funke et al., 2017).

In addition to dominant resistance genes, potential sources of virus resistance are plant genes encoding proviral factors required by the virus to complete its entire infection cycle. This class of host genes is known as susceptibility (S) genes. Mutations in S genes that preclude the virus’s ability to use them for its infection purpose act as virus-resistance genes. In nature, the virus-resistant trait of a mutated susceptibility gene usually occurs in homozygosity, thus being genetically recessive.

The eukaryotic translation initiation proteins from the eIF4E family are major resistance factors for RNA viruses, particularly to *Potyviridae* (Truniger and Aranda, 2009). In flowering plants, the eIF4E family comprises three forms, eIF4E, its isoform eIF(iso)4E, and nCBP (new cap-binding protein), and all are 7-methylguanosine triphosphate (m7GTP) cap-binding proteins, although nCBP does not appear to function in canonical translation (Patrick and Browning, 2012; Browning and Bailey-Serres, 2015). Significantly, eIF4E and eIF(iso)4E functions partially overlap, as shown by gene knockout (KO) and RNA silencing experiments (Duprat et al., 2002; Combe et al., 2005; Atarashi et al., 2020; Miroshnichenko et al., 2020).

The eIF4E family of potato, tomato, and pepper consists of four genes, *eIF4E1* and its paralog *eIF4E2*, *eIF(iso)4E*, and *nCBP*. Notably, natural *eIF4E* alleles conferring PVY resistance have been discovered in pepper and tomato (Ruffel et al., 2002, 2005) but not in *S. tuberosum* L. germplasm (Baebler et al., 2020). The differences between an *eIF4E* susceptible and resistant allele are often few (1–5) non-synonymous amino acid substitutions located mainly near the domain involved in m7GTP cap recognition (Poulicard et al., 2016). The interaction between an eIF4E and the VPg of several potyviruses is closely linked to infection success (Charron et al., 2008; Poulicard et al., 2016), although the precise role(s) of eIF4E in potyvirus infection is still under debate (Bastet et al., 2017).

Based on the evidence of eIF4E requirement for PVY infectivity and the partial gene redundancy between eIF4E and eIF(iso)4E for their translational function, mutagenesis experiments were performed to introduce resistance traits in solanaceous crops by knocking out *eIF4E* genes. A tomato *eIF4E1* EMS splicing mutant was immune to the isolate PVY-LE90 but not to PVY-LE84 (Piron et al., 2010), while the double *eIF4E1/eIF4E2* mutant, although showing a broader PVY resistance, has developmental defects (Gauffier et al., 2016). Similarly, tobacco *eIF4E1* EMS-KO lines were PVY resistant (Julio et al., 2015; Zhao et al., 2020). Conversely, no *eIF4E* KO potatoes were produced, and our knowledge on eIF4E involvement in PVY resistance comes from two opposite transgenic approaches based on eIF4E1: (a) silencing of the native susceptible *eIF4E1*/2 (Miroshnichenko et al., 2020); and (b) overexpression of a resistant *eIF4E1* allele from other *Solanaceae* species (Cavatorta et al., 2011; Duan et al., 2012; Zhang C. et al., 2021) or a potato *eIF4E1* mimicking pepper and tomato resistant alleles (Cavatorta et al., 2011; Arcibal et al., 2016; Gutierrez Sanchez et al., 2020). While silencing of *eIF4E* expression partially mimics the effect of a KO mutant, a more complex and not yet well-understood mechanism underlines the resistance gained through overexpression (Cavatorta et al., 2011; Duan et al., 2012; Gutierrez Sanchez et al., 2020; Zhang C. et al., 2021).

The advent of CRISPR-Cas editing tools has revolutionized our ability to specifically modify plant genomes, boosting our knowledge of genes function and resulting in crops with improved characters (Zhu et al., 2020; Zhang D. et al., 2021). In particular, editing the eIF4E gene family has recently been exploited to confer virus resistance in crops. In cucumber, *eIF4E* KO confers resistance to the potyviruses zucchini yellow mosaic virus and papaya ringspot mosaic virus-W (Chandrasekaran et al., 2016), whereas in tomato it induces a reduced accumulation of PVY^N^ but not PVY^O^ (Atarashi et al., 2020). Similarly, *eIF4E1* editing in the tomato cv. MicroTom confers resistance to pepper mottle virus but not to tobacco etch virus (Yoon et al., 2020). In addition to the use of *eIF4E1*, *eIF4E2* KO has been shown to reduce the accumulation of some isolates of pepper veinal mottle virus in cherry tomato (Kuroiwa et al., 2022), while editing cassava *nCBP1* and *nCBP2* was shown to reduce the severity and incidence of cassava brown streak disease symptoms (Gomez et al., 2019). Thus, depending on the crop and the selected eIF4E gene, resistance to some potyviruses can be introduced.

In this paper, we investigated the impact of *eIF4E1* gene disruption on the ability to broaden the PVY resistance spectrum of the elite tetraploid potato cv. Desirée.^2^ The cv. Desirée is resistant to PVY^O^ but not to PVY^NTN^, among others (Singh et al., 2008; Jones and Vincent, 2018). Protoplasts were transiently transfected with a CRISPR-Cas9 construct targeting *eIF4E1*. After a double plant-transfection regeneration process, *eIF4E1* KO potato plants were obtained. Expression data show that knockout of the *eIF4E1* gene does not alter the mRNA steady-state levels of the other members of the potato eIF4E gene family. According to the recessive resistance character of *eIF4E1*, only plants mutated in all four *eIF4E1* copies were partially resistant to PVY^NTN^. The data demonstrate that *eIF4E1* editing can be profitably used to extend the virus resistance spectrum of elite potato cultivars.

## Materials and Methods

### Plant Material and Potato Virus Y Strain

Nodal stem explants with axillary buds were collected from sprouted certified potato tubers of cv. Desirée (NAK; plantenpaspoort zp-d2/a6/a13 model 2 643.454.408).^3^ Explants sterilization and *in vitro* culture were performed as described in Tavazza and Ancora (1986).

PVY Pa36 was kindly provided by Dr. Massimo Turina and Dr. Marina Ciuffo (Istituto per la Protezione Sostenibile delle Piante IPSP, CNR Torino Italy). PVY Pa36 is an NTN recombinant type. In addition, the 5′ genomic region of PVY Pa36 was PCR amplified using the appropriate primers (Supplementary Table 1) and sequenced to identify which NTN recombinant type it belonged to Green et al. (2017). The sequence shows that the P1 genomic region derives from a PVY^N^ isolate; thus, Pa36 is an NTNa recombinant type (Green et al., 2017).

### sgRNA Design and Plasmid Construction

Potato *eIF4E1* sequences were retrieved from the NCBI database and aligned to identify conserved Cas9 targets. RNA-seq data of the potato cv. Desirée^4^ (Ali et al., 2014) were analyzed with the online free software package Galaxy^5^ (Afgan et al., 2016) to evaluate the presence of SNPs in the selected target sequences. Bowtie2 was run using as reference gene the *eIF4E1* sequence FN666436 and the output (BAM file) used to visualize the distribution of the RNA-seq data on the *eIF4E1* gene using the Integrative Genomic Viewer (IGV) software^6^. Routine molecular cloning procedures were followed for plasmid construction. The HBT-pcoCas9-SteIF4E target6 (HBT-pcoCas9-SteIF4E_6) construct was created from the HBT-pcoCas9 plasmid vector (Addgene_52254; Li et al., 2013).

First, the sgRNA expressing cassette was generated by overlapping PCR on the pUC119-gRNA plasmid (Addgene_52255; Li et al., 2013) using appropriate primers (Supplementary Table 1). The first two PCR reactions were assembled using PrimerF1-*Xho*I + PrR1-SteIF4E-2 and PrF2-SteIF4E-2 + PrimerR2, respectively. In the final PCR reaction, products of the above PCRs were mixed and used as templates with PrimerF1-*Xho*I + PrimerR2 primers. The final PCR product was digested with *Xho*I and *Aat*II and cloned into the HBT-pcoCas9 plasmid.

### Protoplasts Isolation, Transfection, and Regeneration of Potato Plants

Leaf mesophyll protoplasts of *S. tuberosum* cv. Desirée were isolated and cultured following the method and media reported by Tavazza and Ancora (1986) with slight modification. Briefly, before enzyme digestion, leaves were preplasmolysed in the culture medium for 0.5–1 h, and then chopped and incubated overnight in the enzyme solution in the dark at 28°C. After filtration through 297 and 88 μm nylon sieves, the filtrate was diluted with an equal volume of W5 solution (154 mM NaCl, 125 mM CaCl_2_, 5 mM KCl and 2 mM MES, pH 5.8) and centrifuged at 70 × *g* for 5 min. The pellet was washed twice with W5 solution and centrifuged at 70 × *g* for 5 min. The harvested protoplasts were resuspended in W5 solution, stored at room temperature (RT), flat, for 2–3 h in the dark. After centrifugation at 70 × *g* for 5 min, protoplasts were resuspended in MMG solution (4 mM MES pH 5.7, 0.4 M mannitol, 15 mM MgCl_2_) to bring the final concentration to 1.6 × 10^5^ protoplasts ml^–1^ as calculated by microscope on a hemocytometer. PEG-mediated transfection was performed according to Yoo et al. (2007) with modifications. Briefly, 1.6 × 10^5^ protoplasts (200 μl) were mixed with 10 μg of HBT-pcoCas9-SteIF4E_6 (20 μl) and an equal volume (220 μl) of 40% w/v PEG 4000 (Duchefa, Haarlem, The Netherlands), mixed gently and incubated at RT for 10 min. Transfection reaction was stopped by adding 7.5 ml of W5 solution to protoplasts solution followed by centrifugation 5 min at 70 × *g*. Protoplasts were resuspended carefully on 1 ml of culture medium (Tavazza and Ancora, 1986) containing 50 μg ml^–1^ of cefotaxime (Claforan), placed in 12-well cell culture plates, and incubated for 72 h at 24°C in the dark. Protoplasts culture and regeneration were carried out as reported by Tavazza and Ancora (1986).

### Detection of CRISPR-Cas9 Potato Mutants

Multiplex PCR-based high-resolution fragment analysis (MHRFA) (Nicolia et al., 2015) was used to screen the potato regenerants. DNA extracted as described in Edwards et al. (1991) was used to assemble four PCR reactions using a 5′ 6-FAM- or VIC-labeled forward primer (eIF4E_LEI_Fw20_Fam or eIF4E_LEI_Fw20_Vic) together with a 5′G stabilized reverse primer (eIF4E_LEI_Rev201G or eIF4E_LEI_Rev246G) (Supplementary Table 1). The PCR conditions were: 94°C 2’, 35 cycles (94°C 30″, 60°C 30″, 72°C 30″) followed by a 30′ final extension at 72°C. The amount and correctness of PCR products was evaluated by agarose gel electrophoresis and pools of four reactions were equally mixed, run on the Applied Biosystems™ 3130xl Genetic Analyzer (Genechron)^7^ and analyzed using the Peak Scanner Software v1.0 (Applied Biosystems^®^). Selected mutants were further investigated by ICE (Hsiau et al., 2019) analyzing the PCR fragments generated by the primers eIF4E_LEI_Fw20 × eIF4E_LEI_Rev246 or eIF4E_LEI_Fw20 × eIF4e_LEI_Rev297 (Supplementary Table 1).

### Gene Expression Analysis of the eIF4E Gene Family

For gene expression analysis, *in vitro* multiplicated wild-type (wt) and *eIF4E1* mutated potato plants were adapted on soil and grown in a growth chamber set with a photoperiod of 16/8 h (light/dark), and 22/25°C (night/day) temperature regime. The third leaf from the apical meristem was sampled from three plants per genotype and after carefully removing the midvein, all leaf lamina samples were immediately frozen in liquid nitrogen. Total RNA was extracted with the RNeasy Plant Mini Kit (QIAGEN) according to manufacturer’s specifications, and 1 μg was used for cDNA synthesis with QuantiTect Reverse Transcription Kit (QIAGEN). RT-qPCR was performed with SYBR Green PCR Master Mix (Applied Biosystems) on ABI Prism 7900HT (Applied Biosystems) according to the manufacturer’s instructions. Assays were performed in triplicate using 0.3 μl of diluted cDNA (1:5) and 300 nM of both primers in a reaction volume of 10 μl in 384 multiwell plates. PCR primers were designed using the Primer-Blast software.^8^ All primers used for RT-qPCR are listed in Supplementary Table 1. Relative quantification values were calculated with the 2^–ΔΔCt^ method (Livak and Schmittgen, 2001) using *eEF1-a* (AB061263) for normalization (Nicot et al., 2005; Joseph et al., 2018). The relative expression levels of target genes in mutant lines were normalized respect to wt. All the RT-qPCR values shown are from six independent biological replicates from two independent experiments. Kruskal–Wallis statistical test was applied to determine significant differences compared to the wt. *P* < 0.05 was considered statistically significant.

### Virus-Resistance Analysis

*In vitro* multiplicated potatoes were adapted and grown in greenhouse with a photoperiod of 16/8 h (light/dark) and 17/21–25°C (night/day) temperature regime. PVY Pa36 was initially multiplicated in *Nicotiana benthamiana* plants and frozen infected leaf stocks were used as inoculum source. Two leaves of each potato plant, 10–15 cm in height, were inoculated with PVY-infected leaf stock grounded in 50 mM phosphate buffer pH 7.0, 5 mM Na_2_SO_3_ and 1% celite. Four independent infection experiments were performed. The upper leaves of plants were sampled at 16–18 and 28–31 days post inoculation (dpi). Infections were assessed by Northern blot analysis or by RT-qPCR. For Northern blot, total nucleic acids (TNA) were extracted from sampled leaves as described in Brunetti et al. (2001); the probe was a DIG labeled PCR fragment obtained with primers PVY-Fw9054 and PVY-Rev9444 (Supplementary Table 1) on a cDNA derived from reverse transcription of TNA extracted from a PVY Pa36 infected *Nicotiana tabacum* plant. RT-qPCR analysis was performed as described above for eIF4E gene family using PVY Univ Fw and PVY Univ Rev primers (Dai et al., 2013) and *eEF1-a* (AB061263) for normalization (Nicot et al., 2005; Joseph et al., 2018); primers are listed in Supplementary Table 1. Kruskal–Wallis statistical test was applied to determine significant differences compared to the wild type. *P* < 0.05 was considered statistically significant.

### VPg Sequencing

Two micrograms of TNA extracted from PVY Pa36-infected leaves were treated with DNaseI Amplification Grade (Invitrogen) and reverse transcribed with SuperScript III RT (Invitrogen) using random hexamers as recommended by supplier. First strand cDNA was used as template in PCR reactions to amplify the VPg region with Phusion High-Fidelity DNA Polymerase (Thermo Scientific) and primers PVY_NTNab_VPg_Fw and PVY_NTNab_VPg_Rev (Supplementary Table 1). PCR conditions were: 98°C 30″, 35 cycles (98°C 10″, 62°C 20″, 72°C 20″) followed by a 5′ final extension at 72°C. The amplified fragments were sequenced on both strands with the same primers used for PCR.

## Results and Discussion

### Identification of the Target Sequence for Cas9-Mediated *eIF4E1* Knockout

In order to verify whether the eIF4E1 protein is required or essential for PVY infection and to which extent *eIF4E1* Cas9 inactivation can broaden the PVY resistance spectrum, it was crucial to exclude: (a) Cas9 targeting of other genes of the potato eIF4E family, in particular, its paralog *eIF4E2*; (b) possible effects from unwanted truncated proteins acting as dominant-negative mutants.

The BLAST homology search shows that the potato *eIF4E1*, *eIF4E2*, *eIF(iso)4E* and *nCBP* genes are located on chromosome 3, 2, 9, and 10, respectively. Notably, the 5′ end of the *eIF4E1* gene is divergent from the other members of the eIF4 gene family. In addition, previous works showed that N-terminal truncated (aa 38–51) eIF4E proteins retain the ability to bind the m7GTP cap analog and VPg (Monzingo et al., 2007; Ashby et al., 2011; Walter et al., 2019). These data supported the idea that a frameshift mutation or the introduction of a stop codon in the first 114 nt (38 aa) of the gene should not generate a dominant-negative mutant. Thus, we decided to identify Cas9 targets in the 5′ of the *eIF4E1* gene for both aspects mentioned above.

As common tetraploid potato cultivars display a highly heterozygous genome, RNAseq data from cv. Desirée (Ali et al., 2014) were used to assess inter-allelic polymorphism. Several single nucleotide polymorphisms (SNPs) were identified along the *eIF4E1* gene. In particular, four SNPs were present in the first 114 nt, indicating that the Desirée genome possesses at least two *eIF4E1* alleles (Supplementary Figure 1). The first 114 nt of *eIF4E1* contain eight potential (20 nt) Cas9 targets, six in the plus and two in the minus strand. All potential Cas9 targets except target-6 (GTAGACGATGAACTTGAAGA*AGG*) contain at least one SNP (Supplementary Figure 2A) in the so-called crRNA “seed” region (Semenova et al., 2011; Wiedenheft et al., 2011; Jiang et al., 2015).

The target-6 sequence, which has 100% homology among *eIF4E1* alleles, the best score as assessed by CRISPR-P analysis^9^ (Supplementary Figure 2B) and no significant homology with *eIF4E2* sequences (Supplementary Figure 3) was chosen to design the Cas9-guide RNA (gRNA_6).

### Potato *eIF4E1* Knockout Is Compatible With Life

**FIGURE 1:**
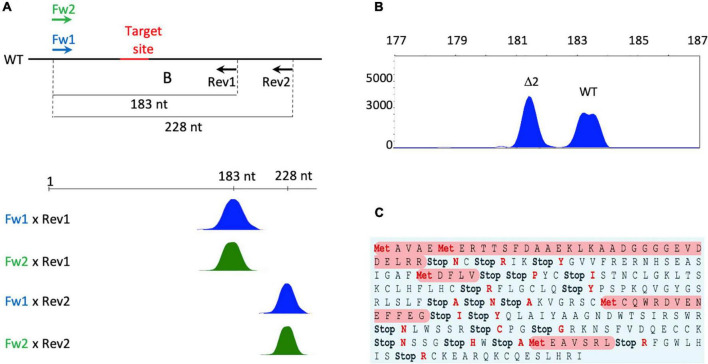
Molecular screening of potato plants regenerated from protoplasts and characterization of line 122 possessing two copies of the *eIF4E1* gene knocked out. **(A)** Schematic representation of high-resolution fragment analysis (MHRFA) to detect Cas9-generated indels. The scheme shows the results expected from wild-type plants. Relative positions of oligonucleotides and CRISPR-Cas9 target site (in red) on Desirée genome. Blue and green represent labeling of forward primers Fw1 and Fw2 with FAM or VIC, respectively. Each peak corresponds to a PCR fragment the size of which is indicated in the upper bar. **(B)** MHRFA of potato line 122. Identification of Δ2 mutations. Each peak corresponds to a PCR fragment the size of which is indicated in the upper bar. The shape of the wild-type peak identifies the presence of two alleles differing in nucleotide polymorphism. **(C)** Potential translation of *eIF4E1* Δ2 alleles. Open reading frames are highlighted in red. Stop, premature stop codons.

High heterozygosity of potatoes coupled with the tetrasomic inheritance (Muthoni et al., 2015) makes outcrossing of the transgene challenging to achieve without affecting the agronomic characteristics of the cultivar. For this reason, to generate *eIF4E1* mutated Desirée plants, Cas9 and gRNA-6 were transiently expressed in protoplasts using the HBT-pcoCas9-SteIF4E_6 construct (Supplementary Figure 4). Four hundred seventy-nine and three hundred fifty-nine shoots were regenerated from two independent transfection experiments. Regenerants were screened for *eIF4E1* mutations by multiplex PCR-based high-resolution fragment analysis (MHRFA) (Figure 1A). In total, 18 mutated clones were identified with an overall mutagenesis frequency of 2.1% (Supplementary Figure 5). However, a marked difference in mutagenesis frequency was observed between the two experiments, 3.3% in the first and 0.6% in the second transfection experiment. The mutation frequency found in the first experiment is in the range with that reported previously (Andersson et al., 2017; Veillet et al., 2019). Thus, the low number of mutated plants obtained in the second experiment seems unrelated to target-6 efficiency. Collectively, eleven clones possessed one mutated *eIF4E1* copy and seven two mutated ones; no plants were mutated in all the four copies. Lines 47, 122, 1,554, and 1,612 were characterized by frameshift mutations in two of the four *eIF4E1* copies (Supplementary Figure 5). Several physical parameters can influence transfection-mediated Cas9 targeting. Andersson et al. (2017) showed that PEG, DNA, protoplasts concentration and DNA incubation time could affect the chance of targeting all four potato gene copies. In particular, they showed, using three different Cas9 targets, that fully knocked out plants could not be recovered with 40% PEG (Andersson et al., 2017). Thus, it could be that our inability to obtain fully knocked out plants in a single round of transfection was derived by the PEG concentration used.

Clone 122, carrying two *eIF4E1* copies characterized by a deletion of two nucleotides (Δ2, Δ2, wt, wt) as bolstered by MHRFA analysis (Figure 1B) and showing a phenotype similar to wt plants was analyzed in more detail. The *eIF4E1* gene region spanning the introduced Cas9 mutation was amplified from genomic DNA and evaluated by ICE analysis. The amplified genomic DNA fragment confirmed that two out of the four copies of the *eIF4E1* gene had a Δ2 mutation (Supplementary Figure 6A). Interestingly, when the same fragment was amplified from the cDNA 99% of the fragments were derived from wt transcripts (Supplementary Figure 6B). Two non-mutually exclusive hypotheses can explain the ICE results: (a) the two copies of the mutated gene were transcribed at a very low level; (b) the *eIF4E1* mutated transcripts were potentially targeted to degradation, possibly by the Nonsense-mediated mRNA Decay (NMD) by the presence of premature termination codons (PTCs) generated by the Δ2 frame-shift (Figure 1C). In plants, similarly to mammals, PTCs, long 3′UTR, and introns > 50–55 nt downstream of TCs can trigger NMD (Shaul, 2015) and these three features are all generated in the Δ2 alleles.

Line 122 which does not contain plasmid derived sequences integrated in its genome was further evaluated for the absence of Cas9-induced mutations in the *eIF4E2* gene. The *eIF4E2* sequence encompassing the potential site (target-6) for Cas9 cutting was amplified and sequenced. No indels or mutations were identified in the *eIF4E2* sequence overlapping the potential Cas9 target-6 site (see below).

Based on the overall evidence, we selected clone 122 for the second round of protoplasts transfection-plant regeneration process. Two hundred sixty-nine shoots were regenerated. MHRFA screening identified 18 plants (C series) with additional mutated *eIF4E1* copies (Supplementary Figure 7). The overall mutagenesis efficiency was 6.7%. Half of the mutated plantlets were mutated in all *eIF4E1* alleles, three of which C14, C29, and C249 were out-of-frame (OF) mutants. Line C249, which showed premature senescence and leaf abscission, was not used in further experiments. The altered phenotype of line C249 appears unrelated to the *eIF4E1* mutation as lines C14 and C29 did not show this phenomenon, thus suggesting somaclonal variations occurring during the regeneration process the cause. Fossi et al. (2019) reported that all potato (Desirée) plants regenerated from protoplasts regardless of showing an altered phenotype have their genomes affected by aneuploidy or structural chromosomal changes.

**FIGURE 2:**
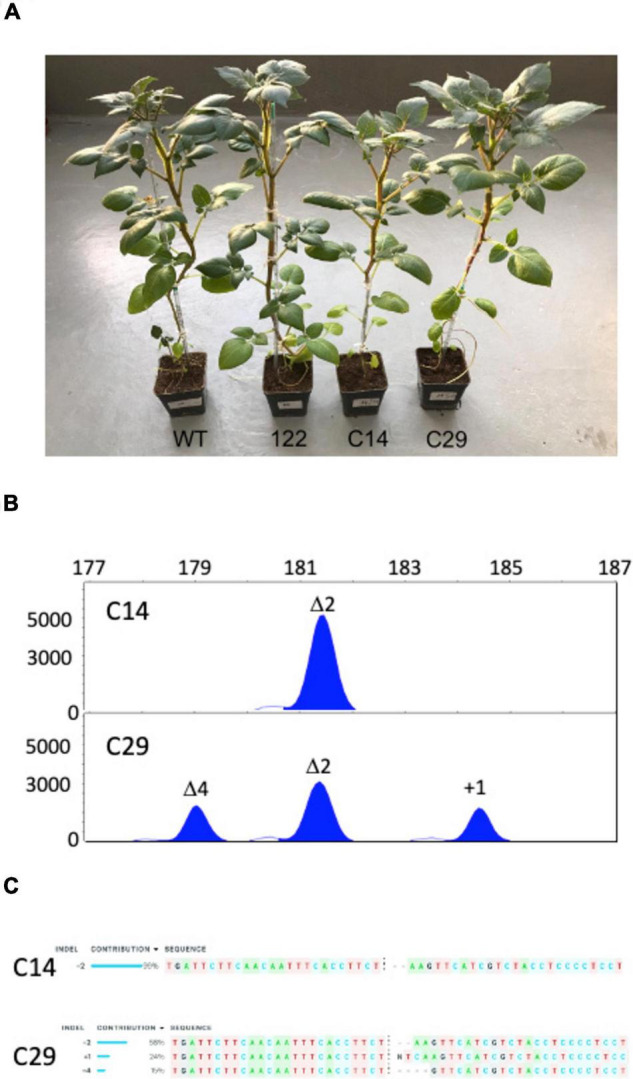
Characterization of the tetraploid potato cv. Desirée plants knockout in the *eIF4E1* gene. **(A)** C14 and C29 potato plant lines knockout in the *eIF4E1* gene together with their progenitor line 122, and wild-type plant. **(B)** High-resolution fragment analysis of the four *eIF4E1* gene copies present in the *eIF4E1* knockout plant lines C14 and C29. **(C)** ICE (https://ice.synthego.com/#/) analysis of PCR fragments amplified from genomic DNA of the C14 and C29 lines.

Lines C14 and C29 (Figure 2A) as bolstered by MHRFA analysis (Figure 2B) were further investigated by ICE analysis (Figure 2C) confirming that the *eIF4E1* genotype of C14 and C29 was (Δ2, Δ2, Δ2, Δ2) and (Δ2, Δ2, Δ4, + 1), respectively, and that, as observed in the progenitor line 122, no mutations were present in the *eIF4E2* sequence overlapping the potential Cas9 target-6 site (Supplementary Figure 8). Thus, only *eIF4E1* and not its paralog *eIF4E2* was mutated in the C14 and C29 lines. Our data show that the knockout of the potato *eIF4E1* gene is compatible with life. This result is in agreement with the data obtained on another *Solanaceae* species, the *Solanum lycopersicon* (Piron et al., 2010).

### A Non-negligible Fraction of the Potato Edited Plants Have Integrated Vector Sequences in Their Genome

Potato genome editing experiments based on the transfection of protoplasts with plasmid DNA can led to stable integration of vector sequences into the plant genome (Clasen et al., 2016; Andersson et al., 2017). The number, size, and area of each eIF4E1 fragment/peak inferred from MHRFA analysis of mutated clones (Supplementary Figure 5 and data not shown) suggest that plasmid DNA integration, if present, was not at the *eIF4E1* target site. To verify if plasmid DNA was integrated into the genome of *eIF4E1* mutated plants, two different regions of the HBT-pcoCas9-SteIF4E_6 construct encompassing Cas9 and gRNA-6 sequences were investigated by PCR analysis. Potato clones possessing two (C13, C28, and C172) and four (C14, C29, and C173) mutated *eIF4E1* copies were initially selected. Only C14 and C29 were transgenic (Supplementary Figure 9). To know to which extent plasmid sequences integration occurred, all 18 mutated potato C clones were analyzed for the presence of HBT-pcoCas9-SteIF4E_6 sequences. PCR analysis showed that in 36% of the potato clones, the vector sequences were integrated into the genome (data not shown). The same percentage of transgenics was observed in plants with three or four *eIF4E1* copies mutated. Thus, the percentage of vector integration does not seem to correlate with the number of mutated *eIF4E1* copies.

Plasmid DNA integration could originate from DNA breaks generated during the transfection-regeneration process (Fossi et al., 2019), Cas9 target activity (Andersson et al., 2017), and micro-homology between plasmid and potato sequences. Our data suggest that DNA integration events were independent of Cas9 activity on the *eIF4E1* target. Differently from our data, Andersson et al. (2017) reported that about 10% (3 out 29) of regenerated potato plants mutated in the granule-bound starch synthase gene had insertion of plasmid DNA sequences into the predicted Cas9 target site; however, no data were reported for random integration of plasmid sequences into the potato genome (Andersson et al., 2017). Similarly, about 10% of potato plants mutated in the amylose-producing *StGBSSI* gene have been shown to have plasmid-derived sequences integrated into their genome (Veillet et al., 2019). In another potato gene editing work, Clasen et al. (2016) reported that approximately 60% of the TALEN-edited potato plants for the vacuolar invertase gene (*VInv*) had vector sequences integrated; these transgenic plants were similarly distributed among those that had one to four edited *VInv* gene copies. Thus, the 36% frequency of vector DNA integration observed here is within the average of those previously reported (Clasen et al., 2016; Andersson et al., 2017; Veillet et al., 2019). Recently, Andersson et al. (2018) showed that transgene-free edited potato plants could be obtained using the Cas9 ribonucleoprotein complex, thus overcoming the bottleneck of vector DNA integration. In our case, the frequency of vector DNA integration was limiting in obtaining transgene-free *eIF4E1* KO plants. Our results together with previous data on potato-edited plants through plasmid-mediated protoplasts transfection suggest that, whenever possible, the use of DNA vectors should be avoided, thus maximizing the output of transgene-free edited potato plants.

### The Disruption of *eIF4E1* Does Not Affect the mRNA Steady-State Levels of Other eIF4E Family Members

The knockout or the knockdown of an eIF4 family member has been shown in some cases to modulate the expression of another gene family member, suggesting the existence of possible regulatory feedback mechanisms (Duprat et al., 2002; Combe et al., 2005; Lellis et al., 2019). An early indication (Duprat et al., 2002) pointed out a possible regulation at the post-transcriptional level. However, an mRNA steady-state level analysis on the consequence of KO of an eIF4 member to the other family members is scarce.

**FIGURE 3:**
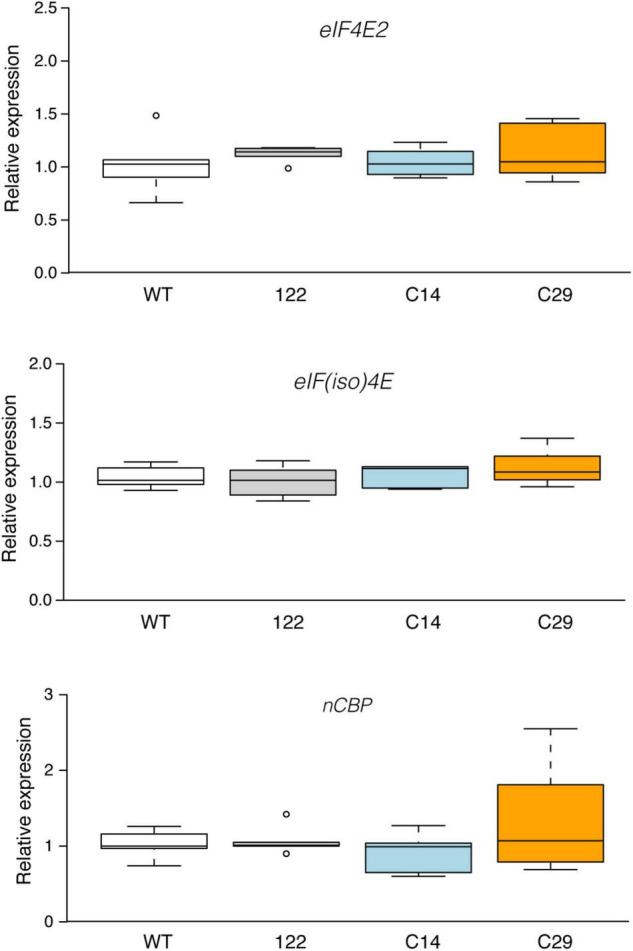
Expression analysis of *eIF4E2*, *eIF(iso)4E*, and *nCBP* genes in *eIF4E1* mutated potato plants. Relative expression levels of the target genes were normalized to the wild-type. Six independent biological replicates from two independent experiments were tested for each genotype. Box plot was generated using the online resource (http://shiny.chemgrid.org/boxplotr/; Spitzer et al., 2014). Center lines show the medians; box limits indicate the 25th and 75th percentiles as determined by R software; whiskers extend 1.5 times the interquartile range from the 25th and 75th percentiles; dots represent outliers. Statistical analyses according to Kruskal-Wallis (*P* < 0.05) reveal no significant differences between wild-type and knockout lines.

Here, RT-qPCR analysis was conducted to evaluate the possible impact of *eIF4E1* disruption on the mRNA steady-state level of *eIF4E2*, *eIF(iso)4E*, and *nCBP*. As shown in Figure 3, wt, 122, C14, and C29 plants accumulate similar amounts of transcripts from each tested gene. Thus, at least under the conditions tested, knockout of the *eIF4E1* gene does not impact the mRNA steady-state levels of the other three potato cap-binding proteins. It remains to be established if a post-transcriptional compensatory regulation mechanism such as that described by Duprat et al. (2002) in the *eIF(iso)4E* Arabidopsis null mutant also applies to the *eIF4E1* potato mutants. The compensatory effect among eIF4s is not only associated with an increased expression of another family member. A double *eIF(iso)4G1* and *eIF(iso)4G2* Arabidopsis KO mutant was shown to accumulate less *eIF(iso)4E* (Lellis et al., 2019). In tobacco, Combe et al. (2005) showed that the reduction of about 60% of the eIF(iso)4E-a and -b proteins, through an antisense approach, led to a compensatory increase in eIF4E levels of approximately 200%. However, the impact of the reduction of eIF(iso)4E-a and -b proteins on the eIF4E mRNA level was not investigated. Conversely, the down-regulation of the *eIF4E* does not trigger a reciprocal increase in *eIFiso4E* levels (Combe et al., 2005). Thus, our data are in line, at least for the lack of variation in *eIF(iso)4E*, with those observed by Combe et al. (2005). To our knowledge, this is the first time that the consequence of *eIF4E1* KO on the mRNA steady-state levels of IF4 family members was investigated.

### *eIF4E1* Knockout Reduces Potato Virus Y Pa36 Multiplication and Ameliorates Virus-Induced Symptoms

Virus resistance, in plants mutated for a gene of the eIF4E family, can range from immunity to a reduction in viral accumulation, and can be restricted to a few viral isolates or show a broad spectrum depending on: (a) the gene(s) of the eIF4E family mutated; (b) the type of mutation introduced; (c) the viral isolates used to challenge the plants (Bastet et al., 2017).

The recessive nature of *eIF4E* mediated resistance predicts that only potato plants mutated in all the *eIF4E1* gene copies can show resistance to PVY. Thus, we expected line 122 to behave like wt plants regard to PVY virus resistance.

**FIGURE 4:**
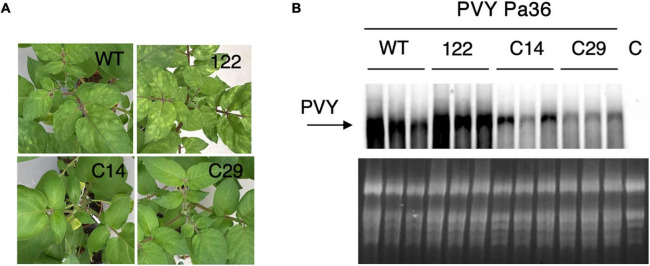
PVY Pa36 multiplication and symptom development are reduced in *eIF4E1* knockout plants. **(A)** Symptoms in PVY Pa36 inoculated plants at 28 dpi. **(B)** Northern blot of TNA extracted from PVY Pa36 inoculated plants at 28 dpi. C, uninoculated wild-type plant.

To initially test the degree of virus resistance of *eIF4E1* mutated potatoes, three wt, 122, C14, and C29 plants were mechanically inoculated with an isolate of the PVY^NTNa^ recombinant type, the PVY Pa36. At 28 days post-inoculation (dpi), wt and 122 plants showed mosaic on the upper leaves, and a slight distortion of leaves margin, C14 plants a pale yellowing, and C29 had very mild symptoms (Figure 4A). Total nucleic acids were extracted from each plant and analyzed by Northern blot for the presence of viral genomic RNA (Figure 4B). In agreement with the recessive nature of eIF4E resistance, line 122, the progenitor of C14 and C29, possessing two unmutated copies of the *eIF4E1* gene, was susceptible to PVY Pa36 like the wt plants. Conversely, in both fully mutated *eIF4E1* lines, PVY Pa36 accumulation was reduced, albeit with some plant-to-plant difference in the C14 line (Figure 4B).

**FIGURE 5:**
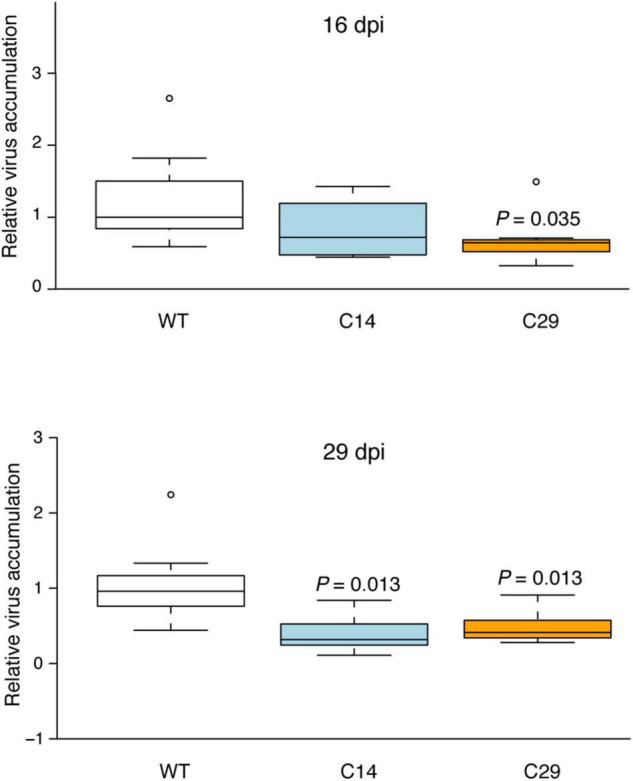
Viral accumulation of PVY Pa36 in *eIF4E1* mutated plants at 16- and 29-days post-inoculation (dpi). Viral accumulation was quantified by RT-qPCR. Seven plants were tested for each genotype. Box plot was generated using the online resource (http://shiny.chemgrid.org/boxplotr/; Spitzer et al., 2014). Center lines show the medians; box limits indicate the 25th and 75th percentiles as determined by R software; whiskers extend 1.5 times the interquartile range from the 25th and 75th percentiles; dots represent outliers. *P-*value of samples with significant difference from wild-type according to Kruskal-Wallis statistical analyses (*P* < 0.05) is shown above the box.

A further experiment was conducted by challenging seven wt, C14, and C29 plants with PVY Pa36 and analyzing virus accumulation by RT-qPCR at 16 and 29 dpi. At 16 dpi, the wt but not C14 and C29 plants showed initial distortion of leaves margin of the non-inoculated upper leaves. RT-qPCR analysis showed a reduction of virus accumulation (difference in the medians) in both *eIF4E1* mutated lines; in C29 plants, the difference was statistically supported (Figure 5). At 29 dpi, a more pronounced distortion of the leaves margin and some localized yellowing of the leaves were present on the wt plants. Conversely, no apparent symptoms were observed in C14 and C29 plants. Coherently with the previous experiment, C14 and C29 plants accumulated less virus than wt plants (Figure 5), thus supporting the notion that the *eIF4E1* KO plants interfere with virus multiplication and-or spread.

The reduction of virus accumulation in *eIF4E1* mutated plants was further confirmed in two additional experiments (Supplementary Figures 10, 11). Again, only mild symptoms were observed in *eIF4E1* mutated potato plants at the end of the experiments (30–31 dpi).

The adaptation of PVY to eIF4E-mediated resistance in tobacco, pepper, and tomato has been reported to involve mutations in the VPg, most often in its central domain (Moury et al., 2004; Charron et al., 2008; Janzac et al., 2014). We then investigated whether the partial resistance observed was due to a rapid virus evolution to replicate and move in the absence of eIF4E1. For this purpose, TNA extracted from two C14 and C29 plants at 28–30 dpi were used to amplify and sequence the viral genome region encoding the VPg. All four virus sequences were identical to the original PVY Pa36 (Supplementary Figure 12). In addition, leaves sap of PVY Pa36-infected C14 plants at 28 dpi was used to back-inoculate four wt, C14, and C29 plants. Again, only mild symptoms were visible on C14 and C29 plants at the end of the experiment (31 dpi) (Supplementary Figure 13). The absence of mutations in the VPg sequence of viruses isolated from C14 and C29 plants, coupled with the inability to induce resistance-breaking symptoms in the back-inoculation experiment, suggests that the reduced inhibition of PVY Pa36 accumulation is not due to the emergence of viral resistance-breaking genotypes. The overall data show that potato plants KO for the *eIF4E1* are partially resistant to PVY Pa36, an isolate belonging to the PVY^NTNa^ recombinant type (Green et al., 2017). The *eIF4E1* KO lines showed a marked reduction of virus-induced symptoms although the resistance was only partial. It remains to be established whether *eIF4E1* KO can prevent potato tuber necrosis ringspot disease (Beczner et al., 1984).

Our data are in line with those obtained on tomato mutants for the *eIF4E1* gene. A tomato *eIF4E1* frameshift mutant generated by CRISPR-Cas9 editing and encoding the first 144 aa of the protein showed a reduced virus accumulation (Atarashi et al., 2020). Similarly, a tomato *eIF4E1* EMS splicing mutant coding for a truncated eIF4E1 protein impaired in cap-binding activity confers resistance to the PVY-LE90 isolate (Piron et al., 2010). Further experiments with different potyviruses will be needed to understand the breadth of resistance of C14 and C29 lines. Preliminary infection data show that C14 and C29 are susceptible as wt and 122 plants to the potato virus V (Lucioli and Tavazza unpublished results). We did not expect Desirée *eIF4E1* KO plants to have a broad-spectrum of resistance to potyviruses. Indeed, in tomatoes, broad-spectrum resistance to potyviruses has been shown to be hampered by eIF4E gene redundancy (Piron et al., 2010; Mazier et al., 2011; Gauffier et al., 2016; Atarashi et al., 2020). As suggested previously (Gauffier et al., 2016; Bastet et al., 2017), the elective strategy to achieve broad-spectrum resistance to potyviruses should mimic the functional *eIF4E1* resistant allele found in natural diversity such as that found in pepper germplasm. Non-synonymous mutations characterize these *eIF4E1* resistant alleles, most if not all the mutated proteins being impaired in their interaction with potyviral VPg (Wang and Krishnaswamy, 2012). It should be noted that this genetic variability is unknown in potato germplasm and that the obtainment of edited potato plants to mimic resistance alleles found in other *Solanaceae* species could be challenging (Gallois personal communication).

Here we show that the knockout of the potato *eIF4E1* confers a limited resistance to PVY Pa36, suggesting that the eIF4E1 is required but not essential for virus infectivity. It remains to be established whether the reduced accumulation of PVY Pa36 in *eIF4E1* KO potato plants results from a partial impairment of viral replication or spread. Our data support the idea that PVY Pa36 might use another eIF4E protein in addition to eIF4E1 for its infection cycle. Examples of potyviruses whose VPg can interact with both eIF4E1 and eIF4E2 proteins have been described (Mazier et al., 2011). In particular, PVY-LYE84 VPg, unlike that of PVY-LE90, has been shown to interact with both tomato eIF4E1 and eIF4E2 protein (Mazier et al., 2011). Similar results were observed with a PVY^NTN^ isolate in potatoes (Lebedeva et al., 2021). In addition, the requirement of simultaneous mutations of the pepper *eIF4E* and *eIF(iso)4E* genes to confer Chili veinal mottle virus and Pepper veinal mottle virus resistance suggests that these viruses may utilize both proteins (Ruffel et al., 2006; Hwang et al., 2009; Rubio et al., 2009).

## Conclusion

Our data show that the knockout of the *eIF4E1* can broaden the resistance spectrum of the elite cv. Desirée conferred by the *Ny* gene to PVY^O^ and that *eIF4E1* KO does not alter the mRNA steady-state level of other members of the potato eIF4E gene family. The limited resistance observed to PVY Pa36 suggests that this virus could profitably use other proteins of the eIF4E family beyond eIF4E1. A more refined editing strategy based on mimicking natural eIF4E resistance alleles will be required to extend the spectrum of resistance to most potyviruses (Bastet et al., 2017, 2019); in addition, this approach should avoid the potential exposure of eIF4E knocked out plants to threats of potyviruses capable of recruiting alternative eIF4E copies, as recently shown to occur in Arabidopsis (Zafirov et al., 2021). New gene-editing approaches (Bastet et al., 2019; Veillet et al., 2020) and a better understanding of the fine interplay between the eIF4E proteins, VPgs (Lebedeva et al., 2021) and potyviruses infection should help to achieve this goal. At the same time, to preserve the integrity of the elite potato cultivars’ traits, improved transformation-regeneration protocols will be required to minimize somaclonal variations incurring during potato plants regeneration (Fossi et al., 2019).

## Data Availability Statement

The original contributions presented in the study are included in the article/Supplementary Material, further inquiries can be directed to the corresponding author.

## Author Contributions

JB, KF, and MT conceived the idea and designed the strategy. RT performed the potato transfection and regeneration of Cas9 mutants. AL carried out the screening and the molecular analyses of the regenerated plants and performed the PVY infections and northern analyses. SB carried out the RT-qPCR analysis of eIF4E expression and PVY resistance. MT, AL, RT, and SB analyzed the data. MT supervised the work and wrote the manuscript. RT, AL, SB, KF, and JB helped to improve the manuscript. All authors have read and agreed to the published version of the manuscript.

## Conflict of Interest

The authors declare that the research was conducted in the absence of any commercial or financial relationships that could be construed as a potential conflict of interest.

## Publisher’s Note

All claims expressed in this article are solely those of the authors and do not necessarily represent those of their affiliated organizations, or those of the publisher, the editors and the reviewers. Any product that may be evaluated in this article, or claim that may be made by its manufacturer, is not guaranteed or endorsed by the publisher.

## Footnotes

1
http://www.fao.org/faostat/en/#data/QC

2
http://varieties.ahdb.org.uk/varieties/view/Desiree

3
www.nak.nl

4
http://www.ebi.ac.uk/arrayexpress/experiments/E-MTAB-1712/samples/?full=truet

5
https://usegalaxy.org/

6
http://software.broadinstitute.org/software/igv/

7
https://www.genechron.com

8
https://www.ncbi.nlm.nih.gov/tools/primer-blast/

9
http://crispr.hzau.edu.cn/CRISPR2/
