# Supplementary material for: CRISPR-Cas9 Targeting of the eIF4E1 Gene Extends the Potato Virus Y Resistance Spectrum of the Solanum tuberosum L. cv. Desirée
Source: Front Microbiol. 2022 Jun 1;13:873930. doi: 10.3389/fmicb.2022.873930 (PMC9198583; doi:10.3389/fmicb.2022.873930)
Supplement: Supplementary file 1 [file Data_Sheet_1.PDF]

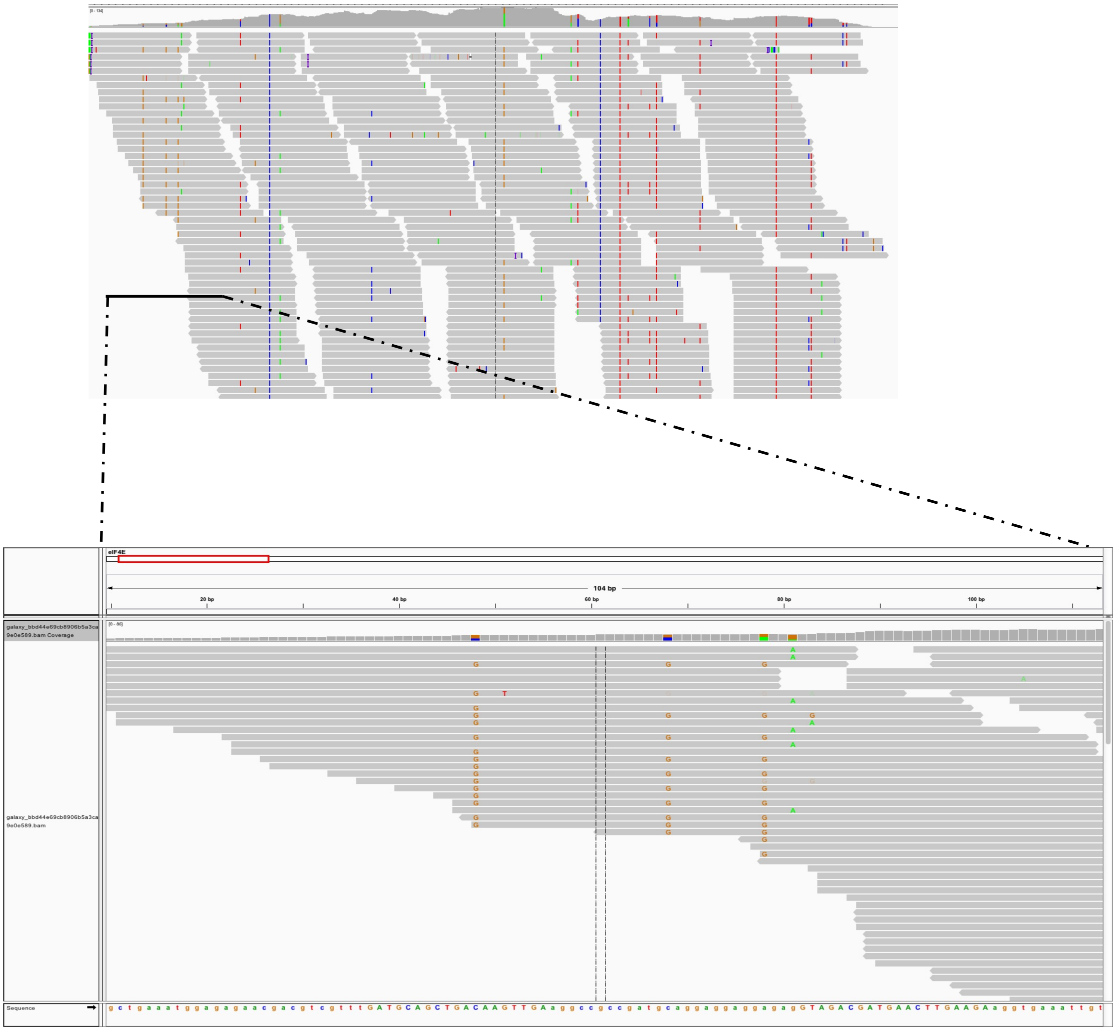

ATGGCAGTAGCTGAAATGGAGAGAACGACGTCGTTTGATGCAGCTGAg/cAAGTTGAAGGCC 60

GCCGATGg/cAGGAGGAGGg/aGAg/aGTAGACGATGAACTTGAAGAAGGTGAAATTGTT 114

Supplementary Figure 1. Analysis of RNAseq data of cv. Desiree (Ali et al., 2014) using the potato *eIF4E1* gene (NCBI #FN666436.1) as reference gene. Orange (G), Green (A), Blue (C), and Red (T) SNPs. Dotted box defines the sequence region (114 nt) chosen for identifying a Cas9 target. In grey, the *eIF4E1* nucleotide sequence that was not considered due to the low coverage in the RNAseq data. In red SNPs present in the different *eIF4E1* Desirée alleles.
