# Supplementary material for: CRISPR-Cas9 Targeting of the eIF4E1 Gene Extends the Potato Virus Y Resistance Spectrum of the Solanum tuberosum L. cv. Desirée
Source: Front Microbiol. 2022 Jun 1;13:873930. doi: 10.3389/fmicb.2022.873930 (PMC9198583; doi:10.3389/fmicb.2022.873930)
Supplement: Supplementary file 2 [file Data_Sheet_2.pdf]

A

Target	Cas9 target sequence	Strand	Nucleotide distance of SNPs from PAM
1	TGATGCAGCTGAgAAGTTGAAGG	+	8
2	AAGTTGAAGGCCGCCGATGcAGG	+	1
3	TTGAAGGCCGCCGATGcAGGAGG	+	4
4	AAGGCCGCCGATGcAGGAGGAGG	+	7
5	CGCCGATGcAGGAGGAGGaGAGG	+	2,12
6	GTAGACGATGAACTTGAAGAAGG	+	
7	CTCtCCTCCTCCTgCATCGGCGG	-	7,17
8	TACCTCtCCTCCTCCTgCATCGG	-	4,14

B

CRISPR-P 2.0

Start Desgin Contact Us Help

Home Submit Design Help News Contact CRISPR-P 1.0 CRISPR-Local

seq_id	sgRNA_id	Score	Sequence	strand	pos	%GC
5' 114nt eIF4E-1	Guide6	0.5841	GTAGACGATGAACTTGAAGAAGG	+	101	40%
5' 114nt eIF4E-1	Guide4	0.1980	AAGGCCGCCGATGCAGGAGGAGG	+	74	70%
5' 114nt eIF4E-1	Guide7	0.1646	CTCTCCTCCTCCTGCATCGGCGG	-	58	70%
5' 114nt eIF4E-1	Guide5	0.1646	CGCCGATGCAGGAGGAGGAGAGG	+	79	70%
5' 114nt eIF4E-1	Guide1	0.1505	TGATGCAGCTGAGAAGTTGAAGG	+	55	45%
5' 114nt eIF4E-1	Guide3	0.1121	TTGAAGGCCGCCGATGCAGGAGG	+	71	65%
5' 114nt eIF4E-1	Guide8	0.0776	TACCTCTCCTCCTCCTGCATCGG	-	61	65%
5' 114nt eIF4E-1	Guide2	0.0041	AAGTTGAAGGCCGCCGATGCAGG	+	68	60%

Supplementary Figure 2. (A) Analysis of SNPs in the Cas9 target sequences in the first 114 nucleotides of the *eIF4E1*. In red nucleotides identifying the location of the SNP. In yellow is highlighted the Cas9 PAM sequence. (B) CRISPR-P score prediction of Cas9 targets. The score range from 0 to 1, the high score predicts the high effectiveness of sgRNA. Presumably best target (score > 0.50), intermediate (0.20 < score < 0.50) low (score < 0.2) CRISPRP http://crispr.hzau.edu.cn/CRISPR2/
