# Supplementary material for: CRISPR-Cas9 Targeting of the eIF4E1 Gene Extends the Potato Virus Y Resistance Spectrum of the Solanum tuberosum L. cv. Desirée
Source: Front Microbiol. 2022 Jun 1;13:873930. doi: 10.3389/fmicb.2022.873930 (PMC9198583; doi:10.3389/fmicb.2022.873930)
Supplement: Supplementary file 3 [file Data_Sheet_3.PDF]

eIF4E-1 (JN831440)
eIF4E-2 (NM_001288408.1)

```
eIF4E1      atggcaacagctgaaatggagagaacgacgtcgtttgatgcagctgagaagttgaaggcc     60
eIF4E2      ------------------------------atggctgatgaactaaacaaagctgcttca     30
                                            *  ***** *    * **        * 

                                   Target-6
eIF4E1      gccgatgcaggaggaggagaggtagacgatgaacttgaagaaggtgaaattgttgaagaa     120
eIF4E2      gaggaatacaaaacgtcatctgtagaagacggaggtgaggaaggagagatcgtaggggaa     90
            *  **      *     *   ***** ** * *  *** ***** ** ** ** *  ***

eIF4E1      tcaaatgatacggcgtcgtatttagggaaagaaatcacagtgaaacatccattggagcat     180
eIF4E2      tcggatgatacggcctcgtttttagggaaacaaatcactatgaaacatccactagaacat     150
            **  ********** **** ********** *******  *********** * ** ***

eIF4E1      tcatggactttttggtttgatagccctattgctaaatctcgacaaactgcttggggaagc     240
eIF4E2      tcttggacattttggttcgataacccttcagggaaatcgaaacaagctgcttggggtagt     210
            ** ***** ******** **** ****   *  *****   **** ********** ** 

eIF4E1      tcacttcgaaatgtctacactttctccactgttgaagatttttggggtgcttacaataat     300
eIF4E2      tccattcgccccatttacaccttctccgctgctgaagatttttggagtgtgtacaacaac     270
            **  ****     * ***** ****** *** ************* ***  ***** ** 

eIF4E1      atccatcacccaagcaagttggttatgggagcagactttcattgttttaagcataaaatt     360
eIF4E2      atccaccacccaagcaagttggccgtgggtgcagacttccattgttttaaaaataaaatt     330
            ***** ****************   **** ******** ***********  ********

eIF4E1      gagccaaagtgggaagatcctgtatgtgccaatggagggacgtggaaaatgaattttttg     420
eIF4E2      gagccaaagtgggaagatcctgtatgtgccaatggagggaagtggacgatgaacttttct     390
            **************************************** *****  ***** ****  

eIF4E1      aagggtaaatctgataccagctggctatatacgctgctggcaatgattggacatcaattc     480
eIF4E2      aggggtaaatctgatacctgctggctgtatacgcttctggcactgattggagagcaattt     450
            * **************** ******* ******** ****** ******** * ***** 

eIF4E1      gatcacggagatgaaatttgtggagcagtcgttagtgtccggtctaagggagaaaaaata     540
eIF4E2      gattatggagatgaaatttgtggagcggttattaatgttcgagttagacaagaaaaaata     510
            *** * ******************** **  *** *** **   **    **********

eIF4E1      gctttgtggaccaagaatgctgcaaatgaaacagctcaggttagcattggtaagcaatgg     600
eIF4E2      gctctgtggaccaggaatgctgccaatgaaacagctcaggtgagcattggtaaacaatgg     570
            *** ********** ******** ***************** *********** ******

eIF4E1      aagcagtttctagattacagcgattcggttggcttcatatttcacgatgatgcaaagagg     660
eIF4E2      aaagagtttctggattacaatgacacaattggctttatatttcatgatgatgcaaagaag     630
            **  ******* *******  **  *  ******* ******** ************* *

eIF4E1      ctcgacagaagtgccaagaatcgttacaccgtatag         696
eIF4E2      cttgacagagctgccaagaatcgttattccgtgtag         666
            ** ******  ***************  **** ***
```

Supplementary Figure 3. Nucleotide sequence homology between *eIF4E1* and *eIF4E2* paralogs. Regions corresponding to the Cas9 target-6 sequences are boxed. PAM sequence is highlighted in yellow
