# Supplementary material for: CRISPR-Cas9 Targeting of the eIF4E1 Gene Extends the Potato Virus Y Resistance Spectrum of the Solanum tuberosum L. cv. Desirée
Source: Front Microbiol. 2022 Jun 1;13:873930. doi: 10.3389/fmicb.2022.873930 (PMC9198583; doi:10.3389/fmicb.2022.873930)
Supplement: Supplementary file 5 [file Data_Sheet_5.PDF]

Potato clone	eIF4E-1 alleles			
47	wt	wt	Δ4	+1
69	wt	wt	wt	+1
92	wt	wt	wt	Δ4
122	wt	wt	Δ2	Δ2
131	wt	wt	wt	+1
148	wt	wt	Δ5	Δ6
149	wt	wt	wt	Δ4
173	wt	wt	wt	Δ4
296	wt	wt	wt	+1
1502	wt	wt	wt	+1
1552	wt	wt	wt	+1
1554	wt	wt	+1	+1
1560	wt	wt	wt	Δ4
1604	wt	wt	wt	+1
1612	wt	wt	Δ2	Δ2
1623	wt	wt	Δ5	Δ6
1642	wt	wt	wt	+1
1762	wt	wt	Δ6	Δ7

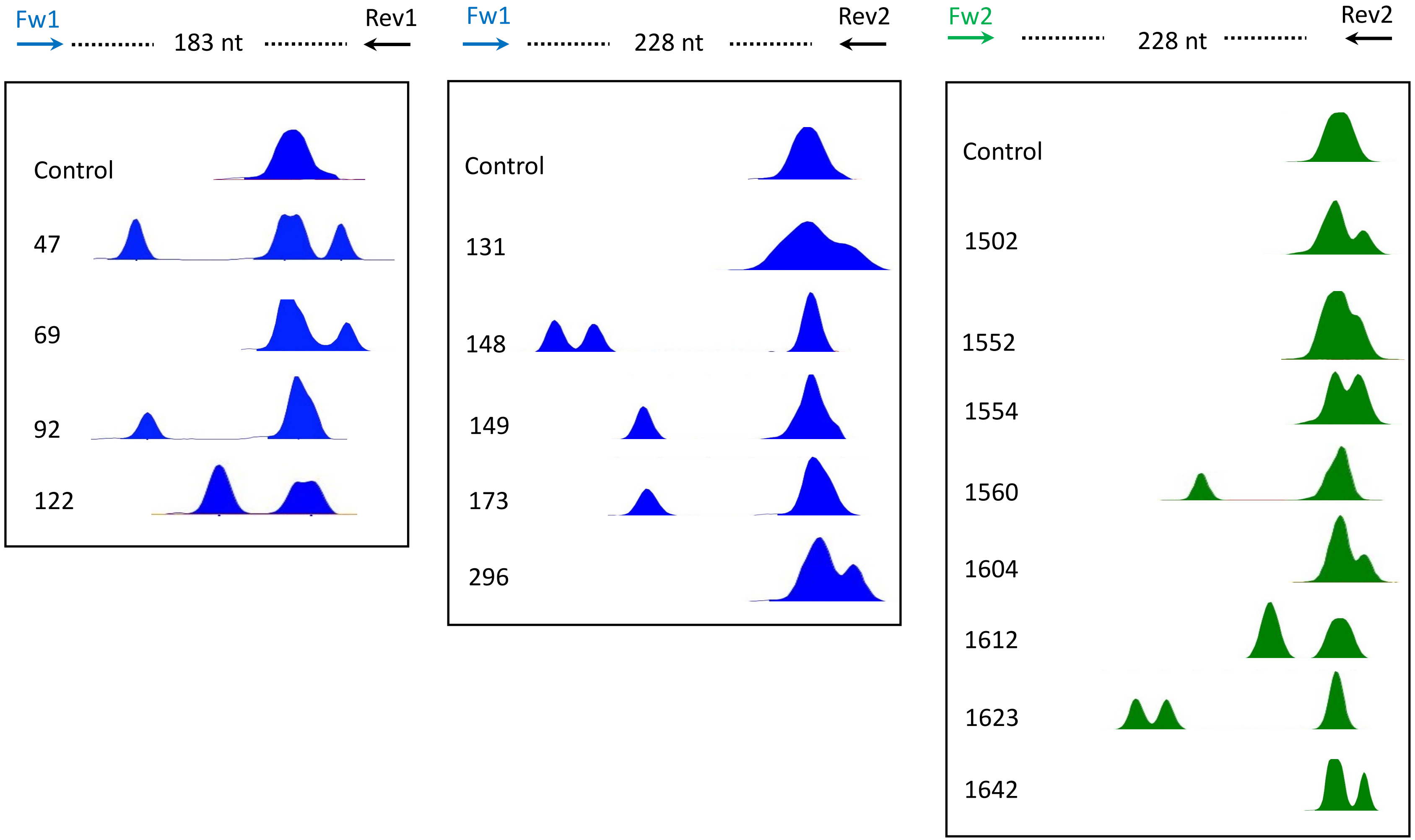

Supplementary Figure 5. Potato cv. Desirée clones possessing at least one mutated *eIF4E1* allele. Light grey: clones carrying two mutated *eIF4E1* alleles. Dark grey: clones carrying two KO *eIF4E1* alleles
