# Supplementary material for: CRISPR-Cas9 Targeting of the eIF4E1 Gene Extends the Potato Virus Y Resistance Spectrum of the Solanum tuberosum L. cv. Desirée
Source: Front Microbiol. 2022 Jun 1;13:873930. doi: 10.3389/fmicb.2022.873930 (PMC9198583; doi:10.3389/fmicb.2022.873930)
Supplement: Supplementary file 6 [file Data_Sheet_6.PDF]

A

ICE analysis of *eIF4E1* DNA

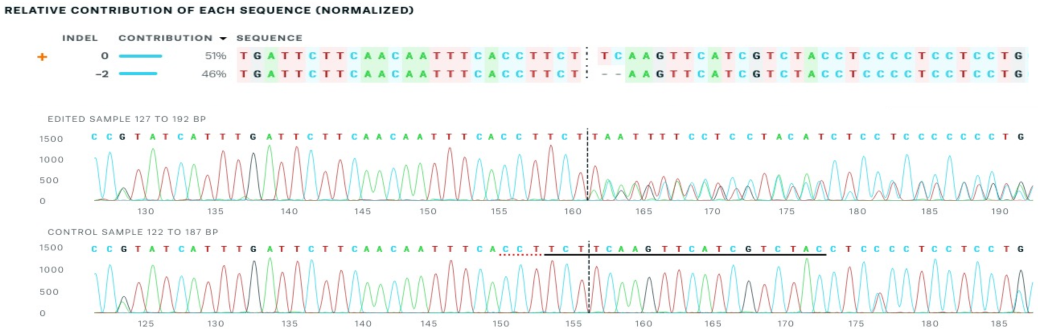

B

ICE analysis of *eIF4E1* cDNA

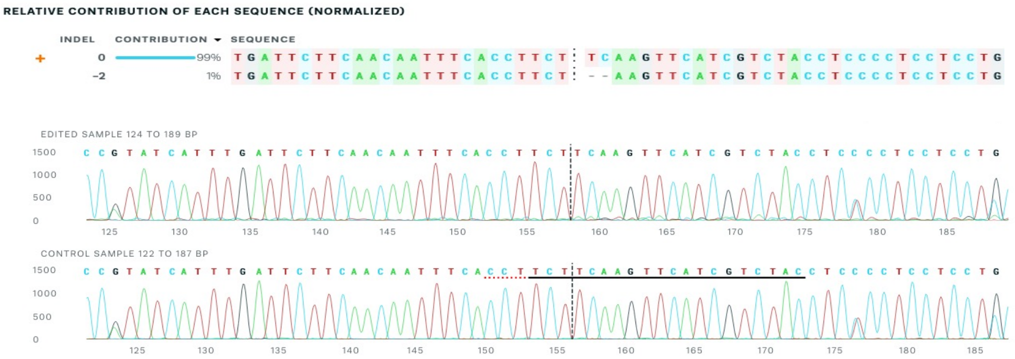

Supplementary Figure 6. (A) ICE analysis of *eIF4E1* genomic DNA reverse sequences spanning the Cas9 target site of plant 122. (B) ICE analysis of *eIF4E1* cDNA reverse sequences spanning the Cas9 target site of plant 122.
