# Supplementary material for: CRISPR-Cas9 Targeting of the eIF4E1 Gene Extends the Potato Virus Y Resistance Spectrum of the Solanum tuberosum L. cv. Desirée
Source: Front Microbiol. 2022 Jun 1;13:873930. doi: 10.3389/fmicb.2022.873930 (PMC9198583; doi:10.3389/fmicb.2022.873930)
Supplement: Supplementary file 7 [file Data_Sheet_7.PDF]

Potato clone	eIF4E-1 alleles			
C14	Δ2	Δ2	Δ2	Δ2
C29	Δ2	Δ2	Δ4	+1
C43	Δ2	Δ2	Δ9	wt
C46	Δ2	Δ2	Δ6	+1
C60	Δ2	Δ2	Δ6	+1
C66	Δ2	Δ2	Δ9	wt
C76	Δ2	Δ2	Δ5	wt
C80	Δ2	Δ2	Δ6	+1
C83	Δ2	Δ2	Δ4	Δ6
C113	Δ2	Δ2	Δ4	wt
C155	Δ2	Δ2	Δ3	wt
C157	Δ2	Δ2	+1	wt
C158	Δ2	Δ2	+1	wt
C173	Δ2	Δ2	Δ4	Δ6
C186	Δ2	Δ2	Δ4	Δ6
C197	Δ2	Δ2	+1	wt
C249	Δ2	Δ2	-1	+1
C256	Δ2	Δ2	Δ4	wt

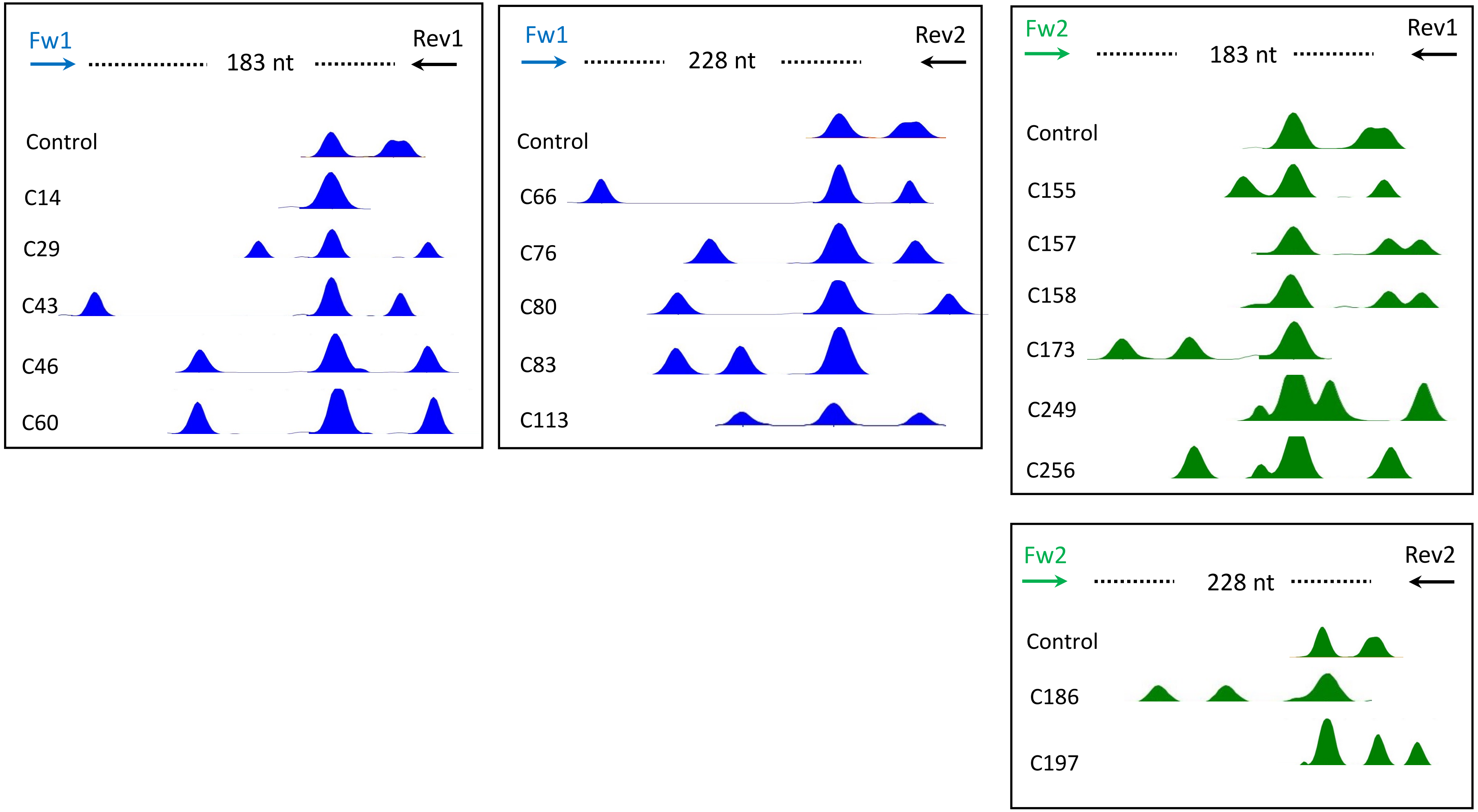

Supplementary Figure 7. Potato cv. Desirée clones possessing at least three mutated *eIF4E1* alleles. Light grey: clones carrying four mutated *eIF4E1* alleles. Dark grey: *eIF4E1* KO clones
