# Supplementary material for: CRISPR-Cas9 Targeting of the eIF4E1 Gene Extends the Potato Virus Y Resistance Spectrum of the Solanum tuberosum L. cv. Desirée
Source: Front Microbiol. 2022 Jun 1;13:873930. doi: 10.3389/fmicb.2022.873930 (PMC9198583; doi:10.3389/fmicb.2022.873930)
Supplement: Supplementary file 8 [file Data_Sheet_8.pdf]

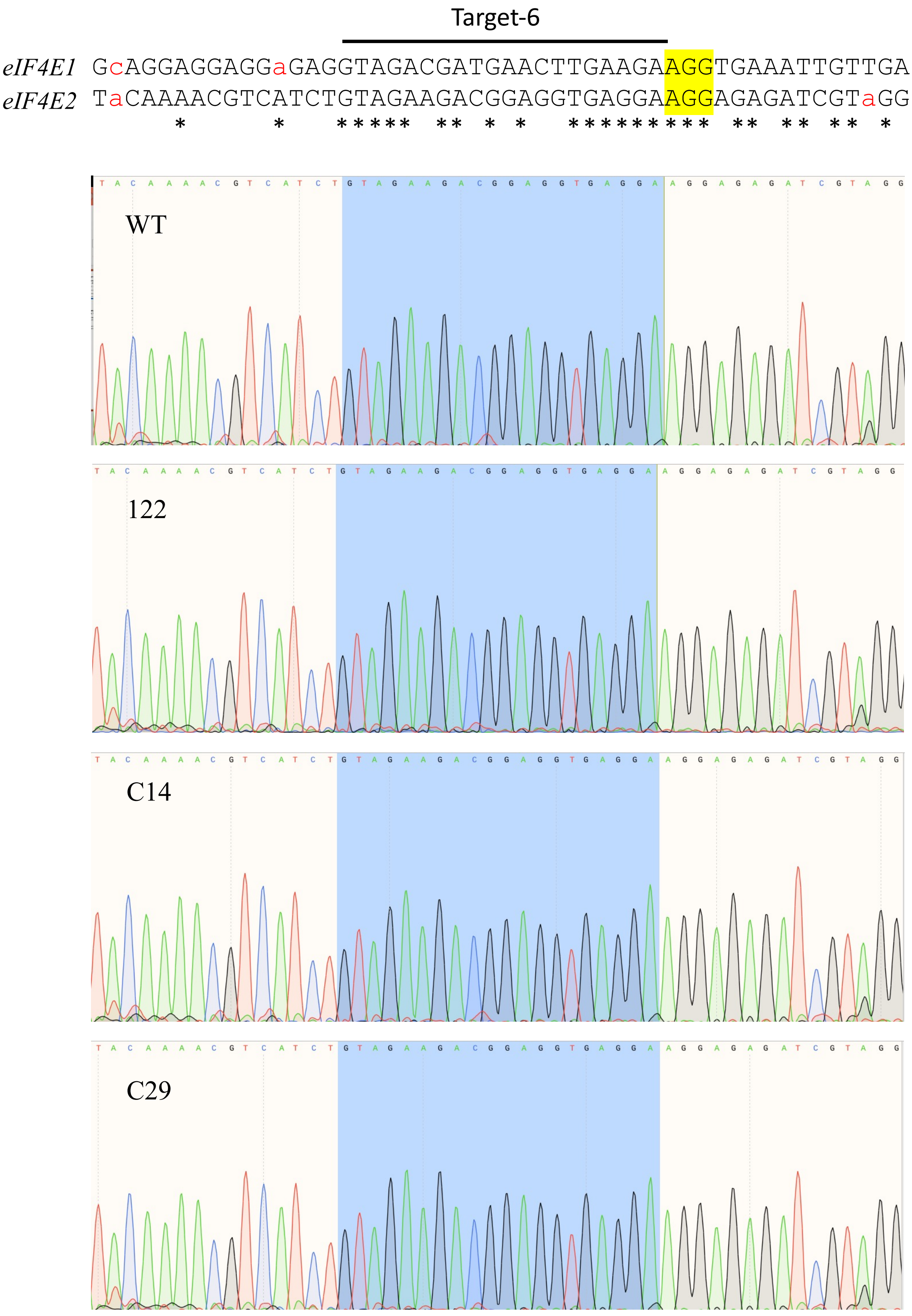

Supplementary Figure 8. Electropherograms of the *eIF4E2* region encompassing the potential Cas9 target-6 in wild-type, 122, C14 and C29 potato plants. Alignment of *eIF4E1* and *eIF4E2* sequences. Red lower cases indicate SNPs present in the *eIF4E1* and *eIF4E2* Desirée alleles. Nucleotides highlighted in yellow identify Cas9 PAM sequence
