# Supplementary material for: CRISPR-Cas9 Targeting of the eIF4E1 Gene Extends the Potato Virus Y Resistance Spectrum of the Solanum tuberosum L. cv. Desirée
Source: Front Microbiol. 2022 Jun 1;13:873930. doi: 10.3389/fmicb.2022.873930 (PMC9198583; doi:10.3389/fmicb.2022.873930)
Supplement: Supplementary file 9 [file Data_Sheet_9.pdf]

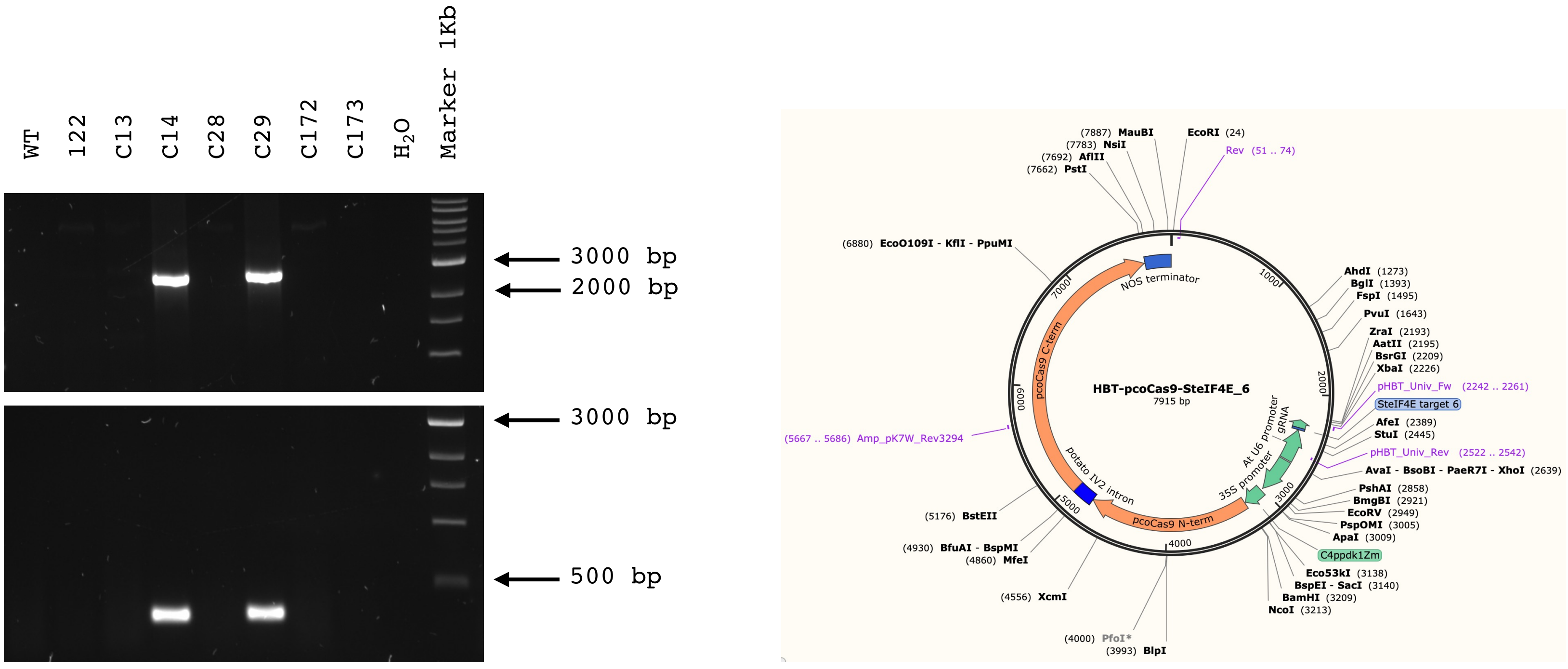

Supplementary Figure 9. Analysis of the presence of plasmid-derived sequences in potato plants derived from the second-round of protoplasts transfection. Upper gel: PCR with Rev x Amp_pK7W_Rev3294 (expected size 2303bp). Lower gel: PCR with pHBT_Univ_ Fw x pHBT_Univ_Rev (expected size 300bp). Potato lines C13, C28 and C172 have the same *eIF4E1* genotype as line 122 (Δ2, Δ2, WT, WT). Potato lines C14 (Δ2, Δ2, Δ2, Δ2), C29 (Δ2, Δ2, Δ4, +1), C173 (Δ2, Δ2, Δ4, Δ6) all have the four *eIF4E1* mutated alleles but only C14 and C29 have the *eIF4E1* fully knocked-out.
