# Supplementary material for: CRISPR-Cas9 Targeting of the eIF4E1 Gene Extends the Potato Virus Y Resistance Spectrum of the Solanum tuberosum L. cv. Desirée
Source: Front Microbiol. 2022 Jun 1;13:873930. doi: 10.3389/fmicb.2022.873930 (PMC9198583; doi:10.3389/fmicb.2022.873930)
Supplement: Supplementary file 10 [file Data_Sheet_10.PDF]

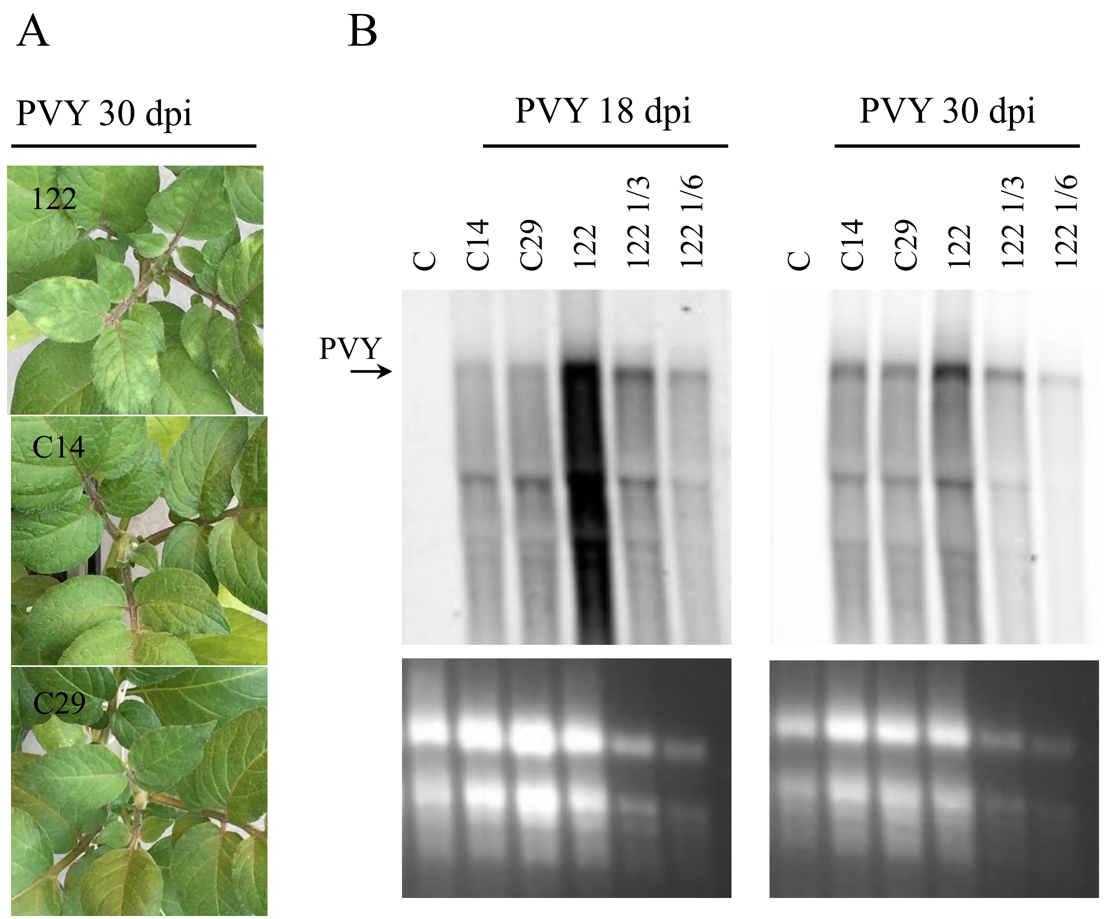

Supplementary Figure 10. Four plants of the control line 122 and five of C14 and C29 were challenged with PVY Pa36 and viral accumulation analyzed by northern blot at 18 and 30 days post infection (dpi)

(A) Plant phenotype at 30 dpi

(B) TNA was extracted from each plant, pooled, and analyzed by northern blot for the presence of the viral RNA at 18 and 30 dpi. C, uninoculated control wild-type plant
