# Supplementary material for: CRISPR-Cas9 Targeting of the eIF4E1 Gene Extends the Potato Virus Y Resistance Spectrum of the Solanum tuberosum L. cv. Desirée
Source: Front Microbiol. 2022 Jun 1;13:873930. doi: 10.3389/fmicb.2022.873930 (PMC9198583; doi:10.3389/fmicb.2022.873930)
Supplement: Supplementary file 11 [file Data_Sheet_11.PDF]

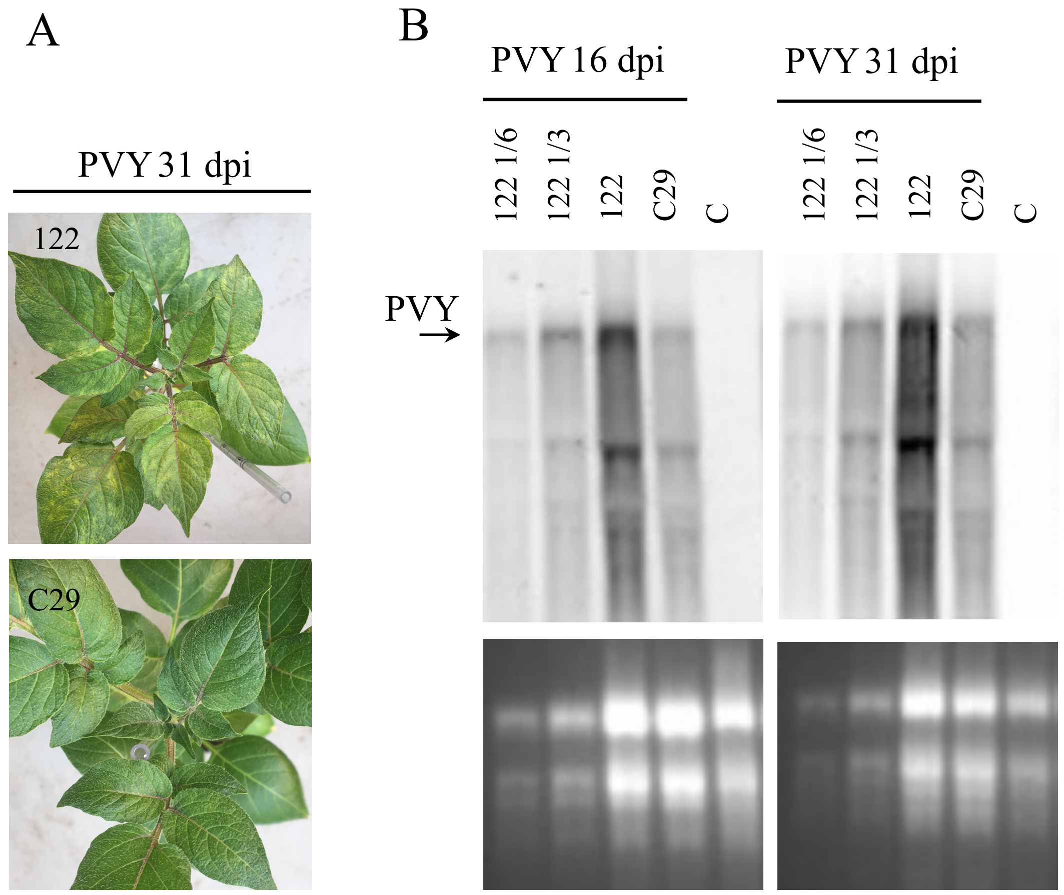

Supplementary Figure 11. Five plants of the control line 122 and C29 were challenged with PVY Pa36 and viral accumulation analyzed by northern blot at 16 and 31 days post inoculation (dpi)
(A) Plant phenotype at 31 dpi
(B) TNA was extracted from each plant, pooled, and analyzed by northern blot for the presence of viral RNA at 16 and 31 dpi. C, control uninoculated wt plant.
