# Supplementary material for: CRISPR-Cas9 Targeting of the eIF4E1 Gene Extends the Potato Virus Y Resistance Spectrum of the Solanum tuberosum L. cv. Desirée
Source: Front Microbiol. 2022 Jun 1;13:873930. doi: 10.3389/fmicb.2022.873930 (PMC9198583; doi:10.3389/fmicb.2022.873930)
Supplement: Supplementary file 12 [file Data_Sheet_12.PDF]

```
VPg_wt_Rev_Reverse         GGGAAAAATAAATCCAAAAGAATTCAAGCCTTGAAGTTTCGCCATGCTCGTGACAAAAGG
VPg_C14_479_Rev_Reverse    GGGAAAAATAAATCCAAAAGAATTCAAGCCTTGAAGTTTCGCCATGCTCGTGACAAAAGG
VPg_C14_477_Rev_Reverse    GGGAAAAATAAATCCAAAAGAATTCAAGCCTTGAAGTTTCGCCATGCTCGTGACAAAAGG
VPg_C29_412_Rev_Reverse    GGGAAAAATAAATCCAAAAGAATTCAAGCCTTGAAGTTTCGCCATGCTCGTGACAAAAGG
VPg_C29_410_Rev_Reverse    GGGAAAAATAAATCCAAAAGAATTCAAGCCTTGAAGTTTCGCCATGCTCGTGACAAAAGG
                           ************************************************************

VPg_wt_Rev_Reverse         GCTGGCTTTGAAATTGACAACAATGATGACACAATAGAGGAATTCTTTGGATCTGCATAC
VPg_C14_479_Rev_Reverse    GCTGGCTTTGAAATTGACAACAATGATGACACAATAGAGGAATTCTTTGGATCTGCATAC
VPg_C14_477_Rev_Reverse    GCTGGCTTTGAAATTGACAACAATGATGACACAATAGAGGAATTCTTTGGATCTGCATAC
VPg_C29_412_Rev_Reverse    GCTGGCTTTGAAATTGACAACAATGATGACACAATAGAGGAATTCTTTGGATCTGCATAC
VPg_C29_410_Rev_Reverse    GCTGGCTTTGAAATTGACAACAATGATGACACAATAGAGGAATTCTTTGGATCTGCATAC
                           ************************************************************

VPg_wt_Rev_Reverse         AGGAAGAAGGGAAAAGGTAAAGGCACCACTGTTGGTATGGGCAAGTCAAGCAGGAGGTTT
VPg_C14_479_Rev_Reverse    AGGAAGAAGGGAAAAGGTAAAGGCACCACTGTTGGTATGGGCAAGTCAAGCAGGAGGTTT
VPg_C14_477_Rev_Reverse    AGGAAGAAGGGAAAAGGTAAAGGCACCACTGTTGGTATGGGCAAGTCAAGCAGGAGGTTT
VPg_C29_412_Rev_Reverse    AGGAAGAAGGGAAAAGGTAAAGGCACCACTGTTGGTATGGGCAAGTCAAGCAGGAGGTTT
VPg_C29_410_Rev_Reverse    AGGAAGAAGGGAAAAGGTAAAGGCACCACTGTTGGTATGGGCAAGTCAAGCAGGAGGTTT
                           ************************************************************

VPg_wt_Rev_Reverse         GTTAATATGTATGGATTTGACCCAACAGAATATTCATTCATCCAGTTCGTTGATCCGCTC
VPg_C14_479_Rev_Reverse    GTTAATATGTATGGATTTGACCCAACAGAATATTCATTCATCCAGTTCGTTGATCCGCTC
VPg_C14_477_Rev_Reverse    GTTAATATGTATGGATTTGACCCAACAGAATATTCATTCATCCAGTTCGTTGATCCGCTC
VPg_C29_412_Rev_Reverse    GTTAATATGTATGGATTTGACCCAACAGAATATTCATTCATCCAGTTCGTTGATCCGCTC
VPg_C29_410_Rev_Reverse    GTTAATATGTATGGATTTGACCCAACAGAATATTCATTCATCCAGTTCGTTGATCCGCTC
                           ************************************************************

VPg_wt_Rev_Reverse         ACTGGAGCTCAAATTGAAGAGAACGTCTATGCTGATATTAGAGACATCCAAGAGCGCTTT
VPg_C14_479_Rev_Reverse    ACTGGAGCTCAAATTGAAGAGAACGTCTATGCTGATATTAGAGACATCCAAGAGCGCTTT
VPg_C14_477_Rev_Reverse    ACTGGAGCTCAAATTGAAGAGAACGTCTATGCTGATATTAGAGACATCCAAGAGCGCTTT
VPg_C29_412_Rev_Reverse    ACTGGAGCTCAAATTGAAGAGAACGTCTATGCTGATATTAGAGACATCCAAGAGCGCTTT
VPg_C29_410_Rev_Reverse    ACTGGAGCTCAAATTGAAGAGAACGTCTATGCTGATATTAGAGACATCCAAGAGCGCTTT
                           ************************************************************

VPg_wt_Rev_Reverse         AGTGATGTCCGCAAGAAAATGGTAGAGGATGATGAAATCGAATTGCAAGCATTGGGCAGC
VPg_C14_479_Rev_Reverse    AGTGATGTCCGCAAGAAAATGGTAGAGGATGATGAAATCGAATTGCAAGCATTGGGCAGC
VPg_C14_477_Rev_Reverse    AGTGATGTCCGCAAGAAAATGGTAGAGGATGATGAAATCGAATTGCAAGCATTGGGCAGC
VPg_C29_412_Rev_Reverse    AGTGATGTCCGCAAGAAAATGGTAGAGGATGATGAAATCGAATTGCAAGCATTGGGCAGC
VPg_C29_410_Rev_Reverse    AGTGATGTCCGCAAGAAAATGGTAGAGGATGATGAAATCGAATTGCAAGCATTGGGCAGC
                           ************************************************************

VPg_wt_Rev_Reverse         AACACAACCATTCATGCTTACTTCAGGAAAGATTGGTCTGACAAGGCTCTAAAAATTGAT
VPg_C14_479_Rev_Reverse    AACACAACCATTCATGCTTACTTCAGGAAAGATTGGTCTGACAAGGCTCTAAAAATTGAT
VPg_C14_477_Rev_Reverse    AACACAACCATTCATGCTTACTTCAGGAAAGATTGGTCTGACAAGGCTCTAAAAATTGAT
VPg_C29_412_Rev_Reverse    AACACAACCATTCATGCTTACTTCAGGAAAGATTGGTCTGACAAGGCTCTAAAAATTGAT
VPg_C29_410_Rev_Reverse    AACACAACCATTCATGCTTACTTCAGGAAAGATTGGTCTGACAAGGCTCTAAAAATTGAT
                           ************************************************************

VPg_wt_Rev_Reverse         TTGATGCCACACAACCCACTCAAAATCTGTGATAAATCGAATGGCATTGCTAAGTTTCCT
VPg_C14_479_Rev_Reverse    TTGATGCCACACAACCCACTCAAAATCTGTGATAAATCGAATGGCATTGCTAAGTTTCCT
VPg_C14_477_Rev_Reverse    TTGATGCCACACAACCCACTCAAAATCTGTGATAAATCGAATGGCATTGCTAAGTTTCCT
VPg_C29_412_Rev_Reverse    TTGATGCCACACAACCCACTCAAAATCTGTGATAAATCGAATGGCATTGCTAAGTTTCCT
VPg_C29_410_Rev_Reverse    TTGATGCCACACAACCCACTCAAAATCTGTGATAAATCGAATGGCATTGCTAAGTTTCCT
                           ************************************************************

VPg_wt_Rev_Reverse         GAAAGAGAACTTGAGTTGAGGCAAACTGGGCCAGCAATAGAGGTTGATGTGAAAGACATT
VPg_C14_479_Rev_Reverse    GAAAGAGAACTTGAGTTGAGGCAAACTGGGCCAGCAATAGAGGTTGATGTGAAAGACATT
VPg_C14_477_Rev_Reverse    GAAAGAGAACTTGAGTTGAGGCAAACTGGGCCAGCAATAGAGGTTGATGTGAAAGACATT
VPg_C29_412_Rev_Reverse    GAAAGAGAACTTGAGTTGAGGCAAACTGGGCCAGCAATAGAGGTTGATGTGAAAGACATT
VPg_C29_410_Rev_Reverse    GAAAGAGAACTTGAGTTGAGGCAAACTGGGCCAGCAATAGAGGTTGATGTGAAAGACATT
                           ************************************************************

VPg_wt_Rev_Reverse         CCAAAACAGGAAGTGGAGCATGAA
VPg_C14_479_Rev_Reverse    CCAAAACAGGAAGTGGAGCATGAA
VPg_C14_477_Rev_Reverse    CCAAAACAGGAAGTGGAGCATGAA
VPg_C29_412_Rev_Reverse    CCAAAACAGGAAGTGGAGCATGAA
VPg_C29_410_Rev_Reverse    CCAAAACAGGAAGTGGAGCATGAA
                           ************************
```

Supplementary Figure 12. Multiplication of PVY Pa36 in *eIF4E1* KO C14 (analyzed at 28dpi) and C29 plants (analyzed at 30dpi) does not induce selection of mutated VPg sequences.
