# Supplementary material for: CRISPR-Cas9 Targeting of the eIF4E1 Gene Extends the Potato Virus Y Resistance Spectrum of the Solanum tuberosum L. cv. Desirée
Source: Front Microbiol. 2022 Jun 1;13:873930. doi: 10.3389/fmicb.2022.873930 (PMC9198583; doi:10.3389/fmicb.2022.873930)
Supplement: Supplementary file 13 [file Data_Sheet_13.PDF]

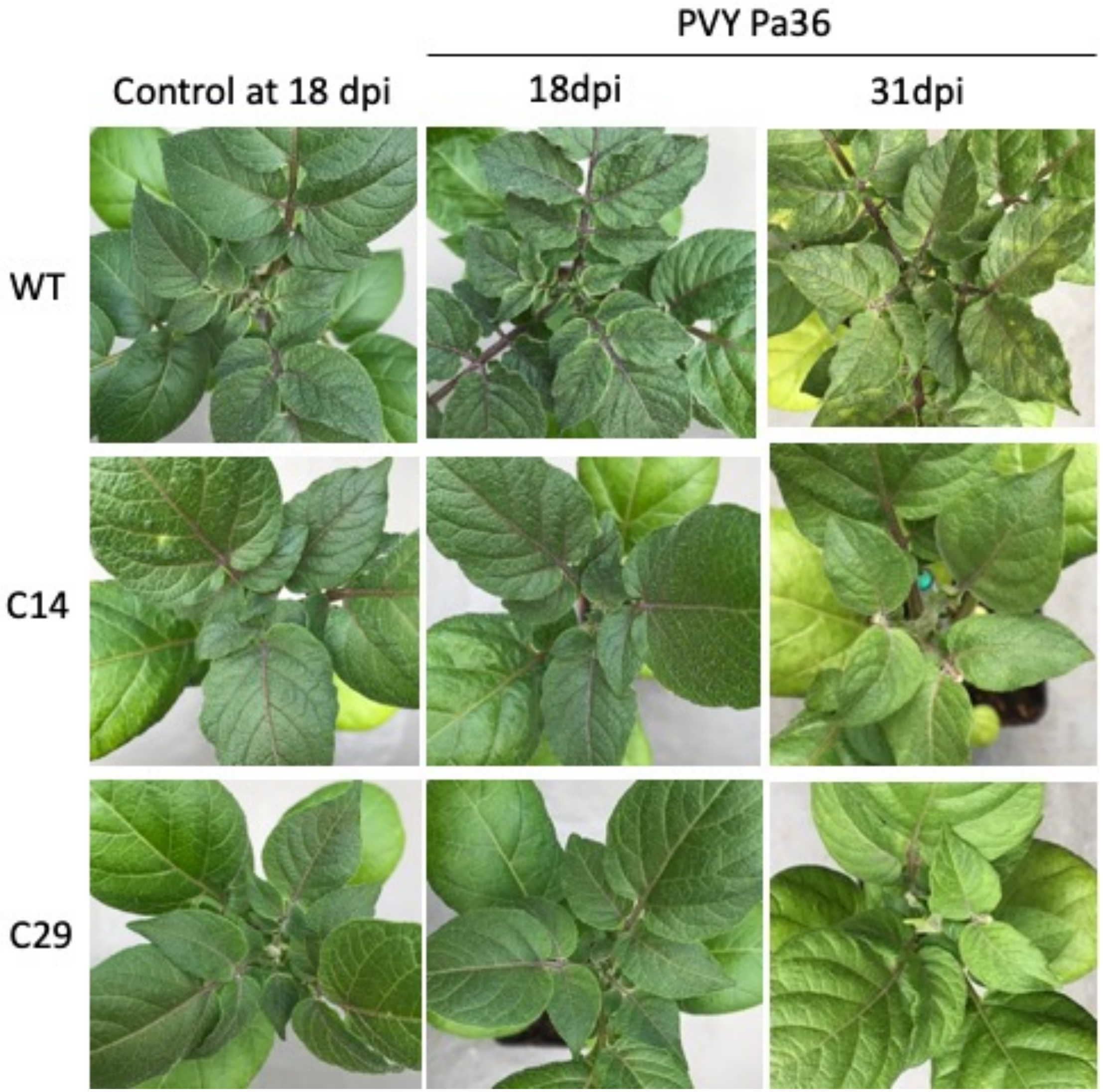

Supplementary Figure 13. Back-inoculation with C14-derived PVY Pa36 infected sap (28 dpi) does not induce a resistance breaking phenotype in C14 and C29 plants
