# Supplementary material for: CRISPR-Cas9 Targeting of the eIF4E1 Gene Extends the Potato Virus Y Resistance Spectrum of the Solanum tuberosum L. cv. Desirée
Source: Front Microbiol. 2022 Jun 1;13:873930. doi: 10.3389/fmicb.2022.873930 (PMC9198583; doi:10.3389/fmicb.2022.873930)
Supplement: Supplementary file 14 [file Table_1.pdf]

CRISPR-Cas9 targeting of the *eIF4E1* gene extends the potato virus Y resistance spectrum of the *Solanum tuberosum* L. cv. Desirée

Supplementary Table 1 Oligonucleotides used

Molecular task	Primer name	Nucleotide sequence	Lenght
eIF4E1 gRNA cassette amplification	PrimerR2	CGCGACGTCTAATGCCAACTTTGTACA	27
	PrimerF1-XhoI	CGCCTCGAGAGAAATCTCAAAATTCCG	27
	PrR1-SteIF4E-2	tcttcaagttcatcgtctacCAATCACTACTTCGTCTCT	39
	PrF2-SteIF4E-2	gtagacgatgaacttgaagaGTTTTAGAGCTAGAAATAGC	40
High resolution fragment analysis	eIF4E_LEI_Fw20_Fam	AGCTGAAATGGAGAGAACGA	20
	eIF4E_LEI_Fw20_Vic	AGCTGAAATGGAGAGAACGA	20
	eIF4E_LEI_Rev201G	GAAGTCCATGAATGCTCCAATG	22
	eIF4E_LEI_Rev246G	GCCCAAGCAGTTTGTCGAGAT	21
ICE analysis	eIF4E_LEI_Fw20	AGCTGAAATGGAGAGAACGA	20
	eIF4E_LEI_Rev246	CCCAAGCAGTTTGTCGAGAT	20
	eIF4E_LEI_Rev297	CCCAAAAATCTTCAACAGTGG	21
PCR-DIG probe	PVY-Fw9054	TCTCAGATGTTGCAGAAGCGT	21
	PVY-Rev9444	AAAAGTAGTACAGGAAAAGCCAAAA	25
RT-qPCR eIF4E gene family	eIF4E2 qPCR Fw	CGGAGGTGAGGAAGGAGAGA	20
	eIF4E2 qPCR Rev	CCCTGAAGGGTTATCGAACCA	21
	eIF(iso)4E qPCR Fw	AGCAGCCGCATAAGCTAGAG	20
	eIF(iso)4E qPCR Rev	GGCGCCTTGTTTCGGTTTAG	20
	nCBP qPCR Fw	TGGAAGTGACACCGGAGAAG	20
	nCBP qPCR Rev	GATCCTCGGCGGCTATTGAG	20
	EF1-alfa qPCR Fw	TGACAGGCGTTCAGGTAAGG	20
	EF1-alfa qPCR Rev	TGGTGGGTATTCAGCAAAGGT	21
RT-qPCR PVY Pa36	PVY Univ Fw	CATAGGAGAAACTGAGATGCCAACT	25
	PVY Univ Rev	TGGCGAGGTTCCATTTTCA	19
	EF1-alfa qPCR Fw	TGACAGGCGTTCAGGTAAGG	20
	EF1-alfa qPCR Rev	TGGTGGGTATTCAGCAAAGGT	21
PVY Pa36 P1 genomic region	PVY_NTNab_P1_Fw	CAACATAAGAAAAWCAACGCAAAAAC	26
	PVY_NTNab_P1_Rev	TGTAGGATATCTCATTTGTGCCC	23
PVY Pa36 VPg amplification and sequencing	PVY_NTNab_VPg_Fw	GAGACTGTGTCTCACCAAGGG	20
	PVY_NTNab_VPg_Rev	CCATACATTTCAGACGTTCCATATT	21
eIF4E2 amplification and sequencing	eIF4E2_Fw	AGAGGAATACAAAACGTCATCTGT	24
	eIF4E2_Rev	GGTGTAAATGGGGCGAATGG	20
Search for vector sequences in the *eIF4E1* edited potatoes	Amp_pK7W_Rev3294	AGCACATCGCTAACCTTGCT	20
	Rev	AGCGGATAACAATTTCACACAGGA	24
	pHBT_Univ_Fw	CGACTCGGTGCCACTTTTTC	20
	pHBT_Univ_Rev	ACGTGAGTGTGAGTGAGACTT	21
