# Supplementary material for: CRISPR-Cas9 Targeting of the eIF4E1 Gene Extends the Potato Virus Y Resistance Spectrum of the Solanum tuberosum L. cv. Desirée
Source: Front Microbiol. 2022 Jun 1;13:873930. doi: 10.3389/fmicb.2022.873930 (PMC9198583; doi:10.3389/fmicb.2022.873930)
Supplement: Supplementary file 4 [file Data_Sheet_4.pdf]

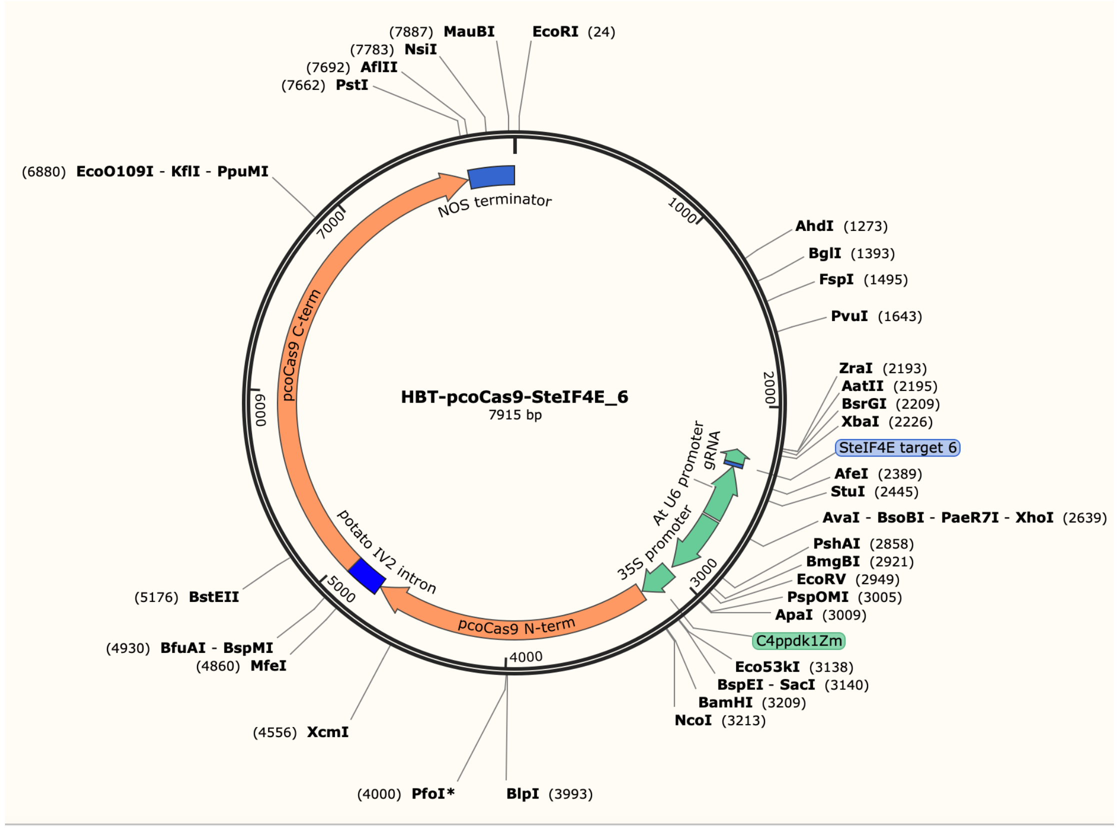

cgatgataagctgtcaaacatgagaattcgtaatcatggtcatagctgtttcctgtgtgaaattgttatccgctcacaattccacacaacatacgagccggaagcataaagtgtaaagcctggggtgcctaatgagtgagctaactcacattaattgcgttgcgctcactgcccgctttcca
gtcgggaaacctgtcgtgccagctgcattaatgaatcggccaacgcgcggggagaggcggtttgcgtattgggcgctcttccgcttcctcgctcactgactcgctgcgctcggtcgttcggctgcggcgagcggtatcagctcactcaaaggcggtaatacggttatccacagaatcagg
ggataacgcaggaaagaacatgtgagcaaaaggccagcaaaaggccaggaaccgtaaaaaggccgcgttgctggcgtttttccataggctccgcccccctgacgagcatcacaaaaatcgacgctcaagtcagaggtggcgaaacccgacaggactataaagataccaggcgttt
ccccctggaagctccctcgtgcgctctcctgttccgaccctgccgcttaccggatacctgtccgcctttctcccttcgggaagcgtggcgctttctcatagctcacgctgtaggtatctcagttcggtgtaggtcgttcgctccaagctgggctgtgtgcacgaaccccccgttcagcccgaccg
ctgcgccttatccggtaactatcgtcttgagtccaacccggtaagacacgacttatcgccactggcagcagccactggtaacaggattagcagagcgaggtatgtaggcggtgctacagagttcttgaagtggtggcctaactacggctacactagaaggacagtatttggtatctgcgc
tctgctgaagccagttaccttcggaaaaagagttggtagctcttgatccggcaaacaaaccaccgctggtagcggtggtttttttgtttgcaagcagcagattacgcgcagaaaaaaaggatctcaagaagatcctttgatcttttctacggggtctgacgctcagtggaacgaaaactca
cgttaagggattttggtcatgagattatcaaaaaggatcttcacctagatccttttaaattaaaaatgaagttttaaatcaatctaaagtatatatgagtaaacttggtctgacagttaccaatgcttaatcagtgaggcacctatctcagcgatctgtctatttcgttcatccatagttgcctg
actccccgtcgtgtagataactacgatacgggagggcttaccatctggccccagtgctgcaatgataccgcgagacccacgctcaccggctccagatttatcagcaataaaccagccagccggaagggccgagcgcagaagtggtcctgcaactttatccgcctccatccagtctattaa
ttgttgccgggaagctagagtaagtagttcgccagttaatagtttgcgcaacgttgttgccattgctacaggcatcgtggtgtcacgctcgtcgtttggtatggcttcattcagctccggttcccaacgatcaaggcgagttacatgatcccccatgttgtgcaaaaaagcggttagctccttc
ggtcctccgatcgttgtcagaagtaagttggccgcagtgttatcactcatggttatggcagcactgcataattctcttactgtcatgccatccgtaagatgcttttctgtgactggtgagtactcaaccaagtcattctgagaatagtgtatgcggcgaccgagttgctcttgcccggcgtcaa
tacgggataataccgcgccacatagcagaactttaaaagtgctcatcattggaaaacgttcttcggggcgaaaactctcaaggatcttaccgctgttgagatccagttcgatgtaacccactcgtgcacccaactgatcttcagcatcttttactttcaccagcgtttctgggtgagcaaaa
acaggaaggcaaaatgccgcaaaaaagggaataagggcgacacggaaatgttgaatactcatactcttcctttttcaatattattgaagcatttatcagggttattgtctcatgagcggatacatatttgaatgtatttagaaaaataaacaaataggggttccgcgcacatttccccga
aaagtgccacctgacgtctaatgccaactttgtacaagaaagctgggtctagaaaaaaagcaccgactcggtgccactttttcaagttgataacggactagccttattttaacttgctatttctagctctaaaactcttcaagttcatcgtctaccaatcactacttcgtctctaaccatatat
aaactcagctgctttctttacctaagcgctgtggtacttttgaagattgttaatagcttaaatgggcctattttagaaaaaggcctatccctcgcttccttctcaagcccatttatattattgaattttttcccaactaaagattttattttcttatgcaagtctcactcacactcacgtaaatttcaa
atataatcgtggtggaactaaaacaatgaccgtctcgtttctacggatcgagattcaaaattgttctgccggaattttgagatttctctcgagaagcttactccaagaatatcaaagatacagtctcagaagaccaaagggctattgagacttttcaacaaagggtaatatcgggaaacct
cctcggattccattgcccagctatctgtcacttcatcaaaaggacagtagaaaaggaaggtggcacctacaaatgccatcattgcgataaaggaaaggctatcgttcaagatgcctctgccgacagtggtcccaaagatggacccccacccacaaggagcatcgtggaaaaagaaga
cgttccaaccacgtcttcaaagcaagtggattgatgtgatatctccactgacgtaagggatgacgcacaatcccactatccttcgccccaagcttgggcccaagcttgggtcgcgccccacggatggtataagaataaaggcattccgcgtgcaggattcacccgttcgcctctcaccttttt
cgctgtactctctcgccacacacaccccctctccagctccgttggagctccggacagcagcaggcgcggggcggtcacgtagtaagcagctctcggctccctctccccttgctccgtggatccatggattacaaggatgatgatgataaggattacaaggatgatgatgataagatggct
ccaaagaagaagagaaaggttggaatccacggagttccagctgctgataagaagtactctatcggacttgacatcggaaccaactctgttggatgggctgttatcaccgatgagtacaaggttccatctaagaagttcaaggttcttggaaacaccgatagacactctatcaagaaga
accttatcggtgctcttcttttcgattctggagagaccgctgaggctaccagattgaagagaaccgctagaagaagatacaccagaagaaagaacagaatctgctaccttcaggaaatcttctctaacgagatggctaaggttgatgattctttcttccacagacttgaggagtctttcctt
gttgaggaggataagaagcacgagagacacccaatcttcggaaacatcgttgatgaggttgcttaccacgagaagtacccaaccatctaccaccttagaaagaagttggttgattctaccgataaggctgatcttagacttatctaccttgctcttgctcacatgatcaagttcagagga
cacttccttatcgagggagaccttaacccagataactctgatgttgataagttgttcatccagcttgttcagacctacaaccagcttttcgaggagaacccaatcaacgcttctggagttgatgctaaggctatcctttctgctagactttctaagtctcgtagacttgagaaccttatcgctca
gcttccaggagagaagaagaacggacttttcggaaaccttatcgctctttctcttggacttaccccaaacttcaagtctaacttcgatcttgctgaggatgctaagttgcagctttctaaggatacctacgatgatgatcttgataaccttcttgctcagatcggagatcagtacgctgatctt
ttccttgctgctaagaacctttctgatgctatccttctttctgacatccttagagttaacaccgagatcaccaaggctccactttctgcttctatgatcaagagatacgatgagcaccaccaggatcttaccctttttgaaggctcttgttagacagcagcttccagagaagtacaaggaaatct
tcttcgatcagtctaagaacggatacgctggatacatcgatggaggagcttctcaggaggagttctacaagttcatcaagccaatccttgagaagatggatggaaccgaggagcttcttgttaagttgaacagagaggatcttcttagaaagcagagaaccttcgataacggatctatc
ccacaccagatccaccttggagagcttcacgctatccttcgtagacaggaggatttctacccattcttgaaggataacagagagaagatcgagaagatccttaccttcagaatcccatactacgttggaccacttgctagaggaaactctcgtttcgcttggatgaccagaaagtctgagg
agaccatcaccccttggaacttcgaggaggtaagtttctgcttctacctttgatatatatataataattatcattaattagtagtaatataatatttcaaatatttttttcaaaataaaagaatgtagtatatagcaattgcttttctgtagtttataagtgtgtatattttaatttataacttttcta
atatatgaccaaaatttgttgatgtgcaggttgttgataagggagcttctgctcagtctttcatcgagagaatgaccaacttcgataagaaccttccaaacgagaaggttcttccaaagcactctcttctttacgagtacttcaccgtttacaacgagcttaccaaggttaagtacgttaccg
agggaatgagaaagccagctttcctttctggagagcagaagaaggctatcgttgatcttcttttcaagaccaacagaaaggttaccgttaagcagttgaaggaggattacttcaagaagatcgagtgcttcgattctgttgaaatctctggagttgaggatagattcaacgcttctcttgg
aacctaccacgatcttttgaagatcatcaaggataaggatttccttgataacgaggagaacgaggacatccttgaggacatcgttcttacccttaccctttttcgaggatagagagatgatcgaggagagactcaagacctacgctcacctttttcgatgataaggttatgaagcagttgaa
gagaagaagatacaccggatggggtagactttctcgtaagttgatcaacggaatcagagataagcagtctggaaagaccatccttgatttcttgaagtctgatggattcgctaacagaaacttcatgcagcttatccacgatgattctcttaccttcaaggaggacatccagaaggctca
ggtttctggacagggagattctcttcacgagcacatcgctaaccttgctggatctccagctatcaagaagggaatccttcagaccgttaaggttgttgatgagcttgttaaggttatgggtagacacaagccagagaacatcgttatcgagatggctagagagaaccagaccacccaga
agggacagaagaactctcgtgagagaatgaagagaatcgaggagggaatcaaggagcttggatctcaaatcttgaaggagcacccagttgagaacacccagcttcagaacgagaagttgtacctttactaccttcagaacggaagagatatgtacgttgatcaggagcttgacatc
aacagactttctgattacgatgttgatcacatcgttccacagtctttcttgaaggatgattctatcgataacaaggttcttacccgttctgataagaacagaggaaagtctgataacgttccatctgaggaggttgttaagaagatgaagaactactggagacagcttcttaacgctaagtt
gatcacccagagaaagttcgataaccttaccaaggctgagagaggaggactttctgagcttgataaggctggattcatcaagagacagcttgttgagaccagacagatcaccaagcacgttgctcagatccttgattctcgtatgaacaccaagtacgatgagaacgataagttgatca
gagaggttaaggttatcaccttgaagtctaagttggtttctgatttcagaaaggatttccagttctacaaggttagagagatcaacaactaccaccacgctcacgatgcttaccttaacgctgttgttggaaccgctcttatcaagaagtacccaaagttggagtctgagttcgtttacgga
gattacaaggtttacgatgttagaaagatgatcgctaagtctgagcaggagatcggaaaggctaccgctaagtacttcttctactctaacatcatgaacttcttcaagaccgagatcacccttgctaacggagagatcagaaagagaccacttatcgagaccaacggagagaccggag
agatcgtttgggataagggaagagatttcgctaccgttagaaaggttctttctatgccacaggttaacatcgttaagaaaaccgaggttcagaccggaggattctctaaggagtctatccttccaaagagaaactctgataagttgatcgctagaaagaaggattgggacccaaagaag
tacggaggattcgattctccaaccgttgcttactctgttcttgttgttgctaaggttgagaagggaaagtctaagaagttgaagtctgttaaggagcttcttggaatcaccatcatggagcgttcttctttcgagaagaacccaatcgatttccttgaggctaagggatacaaggaggttaa
gaaggatcttatcatcaagttgccaaagtactctcttttcgagcttgagaacggaagaaagagaatgcttgcttctgctggagagcttcagaagggaaacgagcttgctcttccatctaagtacgttaacttcctttaccttgcttctcactacgagaagttgaagggatctccagaggata
acgagcagaagcagcttttcgttgagcagcacaagcactaccttgatgagatcatcgagcaaatctctgagttctctaagagagttatccttgctgatgctaaccttgataaggttctttctgcttacaacaagcacagagataagccaatcagagagcaggctgagaacatcatccacc
ttttcacccttaccaaccttggtgctccagctgctttcaagtacttcgataccaccatcgatagaaaaagatacacctctaccaaggaggttcttgatgctacccttatccaccagtctatcaccggactttacgagaccagaatcgatctttctcagcttggaggagataagagaccagctg
ctaccaagaaggctggacaggctaagaagaagaagtgactgcagatcgttcaaacatttggcaataaagtttcttaagattgaatcctgttgccggtcttgcgatgattatcatatatatttctgttgattacgttaagcatgtaataattaacatgtaatgcatgacgttatttatgagatg
ggtttttatgattagagtcccgcaattatacatttaatacgcgatagaaaacaaaatatagcgcgcaaactaggataaattatcgcgcgcggtgtcatctatgttactagatc

Supplementary Figure 4. Sequence and map of HBT-pcoCas9-SteIF4E_6 plasmid used to transfect cv. Desirée protoplasts.
